# The Toronto prehospital hypertonic resuscitation-head injury and multi organ dysfunction trial (TOPHR HIT) - Methods and data collection tools

**DOI:** 10.1186/1745-6215-10-105

**Published:** 2009-11-20

**Authors:** Laurie J Morrison, Sandro B Rizoli, Brian Schwartz, Shawn G Rhind, Merita Simitciu, Tyrone Perreira, Russell MacDonald, Anna Trompeo, Donald T Stuss, Sandra E Black, Alex Kiss, Andrew J Baker

**Affiliations:** 1 Rescu, Keenan Research Centre, Li Ka Shing Knowledge Institute, St Michael's Hospital, 30 Bond St, Toronto Ontario, M5B 1W8, Canada; 2 Department of Medicine, University of Toronto, 1 Kings College Circle, Toronto, Ontario, M5S 1A1, Canada; 3 Sunnybrook Health Sciences Centre, 2075 Bayview Avenue, Toronto, Ontario, M4N 3M5, Canada; 4 Department of Surgery, University of Toronto, 1 Kings College Circle, Toronto Ontario, M5S 1A1, Canada; 5 Sunnybrook Osler Centre for Prehospital Care, 10 Carlson Court, Suite 640, Etobicoke, Ontario, M9W 7K6, Canada; 6 Department of Family and Community Medicine, University of Toronto, 1 Kings College Circle, Toronto Ontario, M5S 1A1, Canada; 7 Defense Research and Development Canada, 1133 Sheppard Avenue West, Toronto Ontario, M3M 3B9, Canada; 8 Department of Anaesthesia, University of Toronto, 1 Kings College Circle, Toronto Ontario, M5S 1A1, Canada; 9 Ornge Transport Medicine, 20 Carlson Court, Suite 400, Toronto Ontario, Canada; 10 University of Turin, Via Po, 53-I-10124 Torino, Italy; 11 Rotman Research Institute, Baycrest, 3560 Bathurst Street, Toronto Ontario, M6A 2E1, Canada; 12 Institute of Clinical and Evaluative Sciences, 2075 Bayview Avenue, Toronto Ontario, M9W 7K6, Canada

## Abstract

**Background:**

Clinical trials evaluating the use of hypertonic saline in the treatment of hypovolemia and head trauma suggest no survival superiority over normal saline; however subgroup analyses suggest there may be a reduction in the inflammatory response and multiorgan failure which may lead to better survival and enhanced neurocognitive function. We describe a feasibility study of randomizing head injured patients to hypertonic saline and dextran vs. normal saline administration in the out of hospital setting.

**Methods/Design:**

This feasibility study employs a randomized, placebo-controlled design evaluating normal saline compared with a single dose of 250 ml of 7.5% hypertonic saline in 6% dextran 70 in the management of traumatic brain injuries. The primary feasibility endpoints of the trial were: 1) baseline survival rates for the treatment and control group to aid in the design of a definitive multicentre trial, 2) randomization compliance rate, 3) ease of protocol implementation in the out-of-hospital setting, and 4) adverse event rate of HSD infusion.

The secondary objectives include measuring the effect of HSD in modulating the immuno-inflammatory response to severe head injury and its effect on modulating the release of neuro-biomarkers into serum; evaluating the role of serum neuro-biomarkers in predicting patient outcome and clinical response to HSD intervention; evaluating effects of HSD on brain atrophy post-injury and neurocognitive and neuropsychological outcomes.

**Discussion:**

We anticipate three aspects of the trial will present challenges to trial success; ethical demands associated with a waiver of consent trial, challenging follow up and comprehensive accurate timely data collection of patient identifiers and clinical or laboratory values. In addition all the data collection tools had to be derived de novo as none existed in the literature.

**Trial registration number:**

NCT00878631

## Background

Hypertonic Saline provides an alternative to large volume time limited resuscitation to restore hemodynamic stability and minimize the posttraumatic organ dysfunction. There is however, no clear clinical evidence supporting the hypothesis that hypertonic saline is the optimal choice for prehospital trauma resuscitation, specifically with respect to brain injury. Most of the current evidence demonstrating anti-inflammatory and immunological properties of hypertonic saline comes from experimental models (animal and isolated cell preparations) and remains untested in humans. A recent meta-analysis demonstrated that hypotensive patients with severe head injury are twice as likely to survive if resuscitated with hypertonic saline in dextran (HSD) compared with isotonic crystalloids [1] In separate clinical trials, HSD resuscitation was associated with a reduced incidence of acute respiratory distress syndrome (ARDS), renal failure and infectious complications [2-4]. Because of the compelling current evidence suggesting the effectiveness of HSD and the known burden of traumatic brain injury (TBI) and multiorgan dysfunction, this question requires direct evaluation.

The application of rigorous clinical trial methodology in the prehospital setting requires overcoming a number of challenges unique to the setting. We conducted a feasibility study prior to implementing a larger randomized clinical efficacy trial. This feasibility study is significantly different from other prehospital trauma trials because it is designed by investigators from multiple disciplines including immunology, neurology, surgery, anaesthesia, neuropsychology and neuroimaging to address aspects of TBI not evaluated previously and employs a randomized clinical trial design and waiver of consent. In addition to the primary study objective of survival at 30 days we plan to evaluate circulating and cellular immunomodulation within the first 24 hours, neurocognitive and neuropsychological testing at 4 and 12 months, and structural damage through MRI scanning up to 4 months post trauma.

We report the methods in detail and have appended the data collection tools and case report forms for this feasibility study. All case report forms and data collection forms were created by the investigators as prior forms did not exist previously in the literature.

## Methods/Design

### Study design

The Toronto Prehospital Hypertonic Resuscitation-Head Injury and Multi Organ Dysfunction Trial (TOPHR HIT) is a randomized, placebo-controlled trial of blunt trauma patients with head injuries. The study compares a group receiving normal saline according to a paramedic's protocol, with a treatment group receiving a single dose 250 ml of 7.5% hypertonic saline in 6% dextran 70 (RescueFlow^® ^BioPhausia AB, Stockholm Sweden). The study was approved by the Canadian Therapeutic Products Directorate Control Number 092523 and registered at http://www.clinical.trials.gov. Inclusion and exclusion criteria are summarized in Additional file 1.

### Study Objective

The primary objective of this study is to report feasibility in accordance with the methodology described by Lancaster and Dodds [5], specifically addressing:

1) baseline survival rates for the treatment and control group to aid in the design of a definitive multicentre trial;

2) randomization compliance rate;

3) ease of protocol implementation in the out-of-hospital setting;

4) adverse event rate of HSD infusion.

The secondary objectives include measuring the effect of HSD in modulating the immuno-inflammatory response to severe head injury and its effect on modulating the release of neuro-biomarkers into serum; evaluating the role of serum neuro-biomarkers in predicting patient outcome and clinical response to HSD intervention; and evaluating effects of HSD on brain atrophy post-injury and neurocognitive and neuropsychological outcomes.

### Randomization

The study employs a block randomization by station derived from a computer-generated random number table. Blocks of two study packages are assigned to each ambulance vehicle/aircraft. Paramedics are blinded to the treatment assigned until package opening. A field logistics research coordinator is responsible for randomization compliance at the vehicle level through daily checks. Compliance at the patient will be verified through the randomization number of the product label and recorded on the data checklist.

### Setting

The trial setting is the city of Toronto with a population of at 2.5 million served by an emergency medical services system employing approximately 430 Advanced Life Support Paramedics, 570 Basic Life Support Paramedics and 3,000 Firefighter first responders under the same medical direction. In addition, the trial includes one Ornge Transport Medicine rotor wing base with two helicopters and 32 critical care flight paramedics providing responses from accident scenes outside the city of Toronto within south central Ontario. Enrolled patients will be directly admitted to two adult designated regional Trauma Centers.

### Intervention Protocol

All patients will be treated according to research medical directives (Additional File 2) based on a standardized provincial out of hospital trauma treatment protocol [6]. The study solution is started within 4 hours of the time the emergency call was received at ambulance dispatch. Samples of peripheral blood will be collected upon emergency department (ED) arrival, 12, 24 and 48 hours after ED arrival from a vein remote from the venous access site for the study infusion. Blood samples will be collected in heparinized and non-additive vacutainers by a single trained lab technician and immediately (within 1-h) shipped to a single designated laboratory for whole blood flow cytometric cellular analyses and measurement of circulating molecular immuno-inflammatory markers. Serum will be frozen for subsequent analyses. Whole blood will be incubated with saturating concentrations of the adhesion (CD62L-FITC, CD11b-PE), degranulation (CD66b-FITC, CD63-PE), apoptotic/necrotic (Annexin V-PE, 7-AAD, Active Caspase-3, CD95-Fas, CD178-FasL) markers, in conjunction with CD14-APC surface staining (to differentiate leukocyte subsets). Results will be acquired on a Becton Dickinson dual-laser (488 nm and 635 nm) BD FACSCalibur flow cytometer using CellQuest software. Serum samples will be analyzed for pro-/anti-inflammatory cytokines (TNF-α, IL-10), soluble leukocyte and endothelial-derived adhesion molecules (sICAM-1, sVCAM-1, sE-selectin, sL-selectin), pro-/anti-apoptotic molecules (sCD95, sCD95L, TNF-RI, sFas/FasL) and neuro-biomarkers of brain injury (S100B, NSE, MBP) (Additional File 3).

Magnetic Resonance Imaging on a 1.5 Tesla General Electric Signa Magnet is planned for completion at 4 months for consenting patients using a standardized protocol (Additional File 4, 5, 6, 7); a 3D 1.3 mm thick, T1-weighted, series (TE 4-2, TR 35, flip angle 35), an interleaved 3 mm thick PD and T2-weighted spin echo sequence (TE 39/80, TR 3000, flip angle 90) (Additional File 6), a gradient echo sequence and Diffusion Tensor Imaging (11 directions) (Additional File 7); tissue segmentation and parcellation including lesion quantification [7-12] using previously published in-house software as well as ANALYZE software [7,13].

### Study outcomes

#### Survival

The primary clinical outcome is survival at 30 days.

Secondary clinical outcomes are survival at 48 hours and to hospital discharge.

#### Neurocognitive Outcomes

At discharge:

• Cerebral Performance Category [14]

At 4 months:

• Functional Independence Measure (FIM)[15]

• Disability Rating Scale (DRS) [16-19]

• Glasgow Outcome Scale (GOS)[16,20,21]

• Glasgow Outcome Scale Extended (GOS Extended)[16,20]

#### Neuropsychological Outcomes

At 4 months:

• Learning and Memory

○California Verbal Learning Test[22]

○Wechsler Memory Scale-Revised Immediate and Delayed Visual Reproduction[23]

○Rey-Osterreith Immediate and Delayed Memory[24]

• Working Memory

○WMS-R Backward Digit Span[23]

• Executive Function

○FAS Verbal Fluency[25]

○Wisconsin Card Sorting Test[26]

○Trail Making Test Parts A & B[27]]

○Stroop Test[28]

○Object Alternation[29]

○WMS-R Forward Digit Span[23]

• Language Function

○Boston Naming Test[27]

○Semantic Fluency[25]

• Visuospatial Function

○Judgment of Line Orientation[30]

○Facial Recognition[30]

• Speed of Processing

○WAIS-R Digit Symbol[31]

○Trail Making Test Part A[27]

• Beck Depression Scale[32].

A focused attention reaction time test[33,34] with varying levels of complexity will be administered at 12 months.

### Structural Outcomes

The structural brain parameters will be evaluated by MRI as described above [7-10] (Additional File 4, 5, 6, 7) and will be measured by a published protocol [8] (Additional File 8, 9). This includes evaluation of:

• Total brain volume and regional volumes (frontal anterior and medial temporal) and medial temporal to be the width [8] (Proton Density T2T1-Weighted) [7]

• Fractional Anistropy (Diffusion Tensor Imaging)

• N-acetyl-aspartate/creatinine ratio (Proton magnetic resonance spectroscopy) [9,10]

• Haemosiderin deposits on gradient echo MRI

• Serum S100B astrocytosis marker of brain injury and C Reactive Protein [35]

### Cellular and Molecular Biomarkers

Whole blood and circulating immuno-inflammatory markers will be measured on hospital arrival and at 12, 24 and 48 hours later

• Total leukocyte count and differential; neutrophil and monocyte surface expression of cellular adhesion molecules and degranulation markers: L-selectin (CD62L) and β_2_-integrin (CD11b) [36]

• Leukocyte apoptotic/necrotic surface and intracellular markers (Annexin V-PE, 7-AAD, Active Caspase-3, Fas (CD95), FasL (CD178) [37-42]

• Soluble pro-/anti-apoptotic molecules (sCD95, sCD95L, TNF-RI, sFas/FasL)[43]

• Leukocyte and endothelial-derived serum soluble adhesion molecule concentrations: intercellular adhesion molecule-1 (sICAM-1), vascular adhesion molecule-1 (sVCAM-1), sE-selectin, sL-selectin [44-46]

• Pro and anti-inflammatory cytokine expression: tumour necrosis factor (TNF)-α, and interleukin (IL)-10 [37,47,48];

• PT/PTT, tissue factor (TF), thrombomodulin (TM), fibrinogen, platelet count and Hg; Ionized calcium, and pH [49]

• Serum sodium, chloride and osmolarity

### Safety and Dependability

The Paramedic Data Checklist (Additional File 10) and both the land and air prehospital (Additional File 11&12) and inhospital (Additional File 13) Case Report Forms explicitly ask whether or not adverse drug reactions with a definite causal relationship to the HSD have occurred. These adverse drug reactions will be predetermined by the Steering Committee based on the literature where possible. (Table 1) The Steering Committee will validate all outcomes and determined adverse events blinded to treatment assignment. This feasibility trial is approved by the Institutional Research Ethics Board.

**Table 1:** Adverse Event Definitions

Event	Expected	Unexpected
**Brain Injury** **(Serious)**	**Demyelination** Any text reference in imaging reports to 'Central Pontine Myelinosis or Pontine Hyperintensity' in any MRI report during hospital stay	**Subarachnoid Hemmorhage** Any text reference in imaging reports of CT of MRI within 24 hours of arrival to Emergency Department to 'Subarachnoid Hemmorhage or SAH' when the first CT scan was normal

**Anaphylactoid Reaction** **(Serious)**	Any text reference in physician notes within the first 24 hours of arrival to Emergency Department to 'anaphylactoid reaction'	

**Prolonged Partial Thromboplastin Time** **(Non serious)**	Normal up to 40 May see up to 85 with HSD	> 100

**International Normalized Ratio** **(Non serious)**	Normal up to 1.5 May see up to 3.0 with HSD	> 3.0

**Serum Sodium** **(Non serious)**	Normal range 135-147 May see up to 158 with HSD	> 160

**Serum Osmolarity** **(Non serious)**	Normal range 310-330 May see up to 350 with HSD without alcohol	> 350 without alcohols

**Serum Chloride** **(Non serious)**	Normal range 96-108 May see up to 145	>150

**Rouleau formation** **(Non serious)**	May be present	

MRI Magnetic Resonance Imaging

CT Computerized Tomography Scan

SAH Subarachnoid Hemmorhage

### Data Management

Paramedics complete a data checklist (Additional File 10) and standardized provincial patient care record. A single paramedic data abstractor will abstract data from the paramedic checklist and patient care record onto a prehospital case report form (Additional File 11). Site specific inhospital research staff will abstract data from the patient's chart daily and completed an inhospital case report form (Additional File 13). In-hospital patient data includes the interpretation of head CT scans employing the Marshall Classification system [50] as well as the Injury Severity Score [51]. Measures of organ dysfunction: Acute Physiologic and Chronic Health Evaluation (APACHE II)[52,53], Sequential Organ Failure Assessment (SOFA) [54] and Multiple Organ Dysfunction Score (MODS)[55] will be evaluated at ICU admission. In addition, SOFA and MODS will be recorded every other day until ICU discharge. Data entry onto a web based (prehospital data) or SAS version 8.0 interface (inhospital and follow up data) will be performed by a single trained data entry specialist.

### Analysis Plan

This is a feasibility study of two year's duration with a projected screening of 130 patients and recruitment of 100. The primary outcome measure, change in the proportion surviving 30 days post trauma between the treatment and control groups, will be analyzed by means of a generalized linear mixed model which included covariates of interest. The two groups are not expected to be different at baseline, and it is hypothesized that the treatment group would show a 20% decrease in mortality at 30 days post trauma whereas the control group would remain unchanged. To derive a reduced subset of risk factors for the predictor models, acceptable model building strategies are planned (i.e. dropping variables showing extremely small variability and those found to be highly correlated (i.e. correlation coefficient ≥0.8)). Treatment and time factors will be analyzed using a generalized linear mixed models of the secondary outcome repeated measures: the change in mortality after discharge over time as well as the neurocognitive and neuropsychological outcome summary scores. For secondary outcome analyses, the alpha will be adjusted to account for multiple comparisons. Where there is more than one measure for a neuropsychological test (e.g., processing speed, working memory, visuospatial, language, verbal memory, non-verbal memory - with the exception of executive processes, since different measures relate to different brain regions), a composite or prototypical measure will be derived from group comparison. Serial changes in all secondary immune modulation measures, neuro-biomarkers, neurocognitive and neuropsychological outcomes, and structural imaging measures will be evaluated by two-way ANOVA for repeated measures. All analysis will be conducted blinded to treatment assignment.

### Ethical Considerations

We received waiver of consent approval in accordance with the Tri-Council Policy Agreement http://www.pre.ethics.gc.ca/english/policystatement/policystatement.cfm. We will seek consent from the surviving patients or the families of non survivors to analyze the blood results of all randomized patients. An itemized consent will be used where appropriate to record simultaneously consent for all follow up tests and evaluation of surviving patients.

## Discussion

The metaanalysis by Wade et al[1] suggested TBI was the cohort of patients most likely to demonstrate significant changes with HSD resuscitation. This study is designed to test the feasibility of randomizing patient in the prehospital setting to this cohort of patients and explore through additional tests, the important immunological, serum markers, structural changes and neuropsychological and neurocognitive outcomes in addition to the traditional survival outcomes.

We anticipate three aspects of the trial will present challenges to trial success; ethical demands, challenging follow up and comprehensive accurate timely data collection of patient identifiers and clinical or laboratory values. The ethical considerations are probably the most challenging as we will have to secure consent to analyze the blood of non survivors from families who have suffered an unexpected and devastating loss through trauma.

Additionally, we are concerned that many patients will be lost to follow up as individuals who survive TBI may be unable to return to their previous address or place of occupation and are very dependent on family. If important links to the family are not established during the first hospitalization and maintained post discharge we may be unable to find all the patients. In addition, the structural and neurocognitive and neuro psychological tests require a return visit to hospital. This may place additional demands on the family and care giver who may be overwhelmed given the functionality of some traumatic brain patients.

Finally, we anticipate two types of patients will provide unique challenges to data collection: those who die in the Emergency Department and those who met the Glasgow Coma Scale criteria for enrolment however this finding was attributed to drugs or alcohol rather than brain injury and they were discharged from the Emergency Department within hours of the accident. Strategies are in place to address these issues a priori in advance of the trial. Those who die soon after arrival in the Emergency Department often require the coroner's intervention and traditionally the charts are instantly sequestered by the coroner's staff. They commonly are unidentified and therefore it will be hard to find their health care advocate who can consent to using their blood and outcome data up until their death. We hope to overcome this with an agreement in principle with the provincial coroner to allow access to the chart at the coroner's office. We have developed a working practice with the coroner's office to confirm identification and next of kin data at the time of body retrieval

We anticipate the patients who met the GCS eligibility criteria based on alcohol consumption and are discharged relatively quickly from the Emergency Department will be hard to track down. There will be insufficient time to identify them correctly or confirm their demographic information and next of kin prior to discharge and follow up of the non brain injured group will be challenging. We will use a hierarchical approach to find these patients including government health care billing number, police report, Emergency Department report, Ambulance call report, web based telephone listings, newspapers which identify the individual based on the notoriety of the event. In addition, each receiving hospital had an active trauma registry which tracked the patients to conduct a functional evaluation at 30 days post-discharge. The surgical investigators have offered to confirm identification and obtain consent in their outpatient clinics if the patients return for follow up. This may prove to be helpful as many of these patients require forms completed by the physician for return to work, disability, insurance etc.

This study incorporates several unique features which may be helpful to subsequent trials. It is a feasibility design that follows a rigorous published methodology. It evaluates the feasibility of the out of hospital randomization as well as the inhospital data capture and analysis of complicated critical care variables, immunological markers, CT and MRI studies, and neurocognitive evaluation. The data capture forms appended to this document had to be created by the investigators as none could be found in the literature despite numerous studies in a similar cohort of patients. This should save others time and permit a more standardized data capture for future trials should they prove useful when reviewed by our peers. We hope providing the methodology will encourage others to test the feasibility of a randomized controlled trial before implementation in the complicated and costly out of hospital arena.

## Abbreviations

ICU: Intensive Care Unit; CT: Computerized Tomography; MRI: Magnetic Resonance Imaging; All other short forms are written in full with first use.

## Competing interests

All committee members were free of conflict of interest with respect to the study fluid manufacturer, study results and the funding source Defense Research and Development Canada (DRDC). Sandro B. Rizoli was a DRDC grant recipient and Shawn G. Rhind was a DRDC employee. Biophausia Sweden provided the study fluid (RescueFlow) free of charge without obligation to the investigators for the duration of the trial.

## Authors' contributions

LJMchaired the steering committee, designed the study, wrote the protocol drafts, drafted the manuscript, implemented the study. SRconceived the study, participated on the steering committee, helped to design the study, contributed to protocol and manuscript drafts and helped to implement the study, received peer review funding for the project, and participated in the planning of the immuno-inflammatory and serum neuro-biomarkers substudies. BSparticipated on the steering committee, helped to design the study, contributed to protocol and manuscript drafts and helped to implement the study in the land EMS system. SRparticipated on the steering committee, helped to design the study, contributed to protocol and manuscript drafts, directed the immuno-inflammatory and serum neuro-biomarkers substudies. MSparticipated on the steering committee, contributed to protocol and manuscript drafts, coordinated the trial with a focus on long term follow up and consent. TPparticipated on the steering committee, contributed to protocol and manuscript drafts, provided the oversight on trial coordination and implementation. RMparticipated on the steering committee, contributed to protocol and manuscript drafts, and helped to implement the study in the air EMS system.

ATcontributed to manuscript drafts, planned the analysis, set up the data management system under the direction of Alex Kiss. DTSparticipated on the steering committee, helped to design the study, contributed to protocol and manuscript drafts, directed the neurocognitive and neuropsychological outcomes substudy. SBparticipated on the steering committee, helped to design the study, contributed to protocol and manuscript drafts, directed the evaluation of brain atrophy post-injury substudy. AKcontributed to protocol and manuscript drafts planned the analysis, set up the data management system.

ABparticipated on the steering committee, helped to design the study, contributed to protocol and manuscript drafts and helped to implement the study and participated in the planning of the immuno-inflammatory and serum neuro-biomarkers substudies.

## Supplementary Material

Additional file 1 Inclusion and exclusion criteria. Click here for file

Additional file 2 Medical Directives. Click here for file

Additional file 3 Blood laboratory Method. Click here for file

Additional file 4 MRI acquisition parameters. Click here for file

Additional file 5 MRI Scan Parameters. Click here for file

Additional file 6 MRI brain atrophy. Click here for file

Additional file 7 MRI DTI. Click here for file

Additional file 8 MRI MTLT in AD. Click here for file

Additional file 9 MRI DTI in AD. Click here for file

Additional file 10 Paramedic data checklist. Click here for file

Additional file 11 Pre-hospital CRF-LAND. Click here for file

Additional file 12 Pre-hospital CRF-AIR. Click here for file

Additional file 13 In-hospital case report form. Click here for file
