# Supplementary material for: The Toronto prehospital hypertonic resuscitation-head injury and multi organ dysfunction trial (TOPHR HIT) - Methods and data collection tools
Source: Trials. 2009 Nov 20;10:105. doi: 10.1186/1745-6215-10-105 (PMC2788534; doi:10.1186/1745-6215-10-105)
Supplement: Additional file 1 — Inclusion and exclusion criteria. [file 1745-6215-10-105-S1.DOC]

| **Appendix 1.** Inclusion and Exclusion Criteria |
| --- |
| **Inclusion Criteria**   - Age ≥ 16; - Initial assessment of GCS 8 or less; - Blunt traumatic mechanism of injury. |
| **Exclusion Criteria**   - Known pregnancy; - Primary injury penetrating; - VSA prior to randomization; - Previous Intravenous therapy ≥ 50 ml; - Time interval between arrival at scene and intravenous access exceeds four hours; - Amputation above wrist or ankle; - Any burn (thermal, chemical, electrical, radiation) - Suspected hypothermia; - Asphyxia (strangulation, hanging, choking, suffocation, drowning) - Fall from height ≤ 1m or ≤ 5 Stairs |
