# Supplementary material for: The Toronto prehospital hypertonic resuscitation-head injury and multi organ dysfunction trial (TOPHR HIT) - Methods and data collection tools
Source: Trials. 2009 Nov 20;10:105. doi: 10.1186/1745-6215-10-105 (PMC2788534; doi:10.1186/1745-6215-10-105)
Supplement: Additional file 2 — Medical Directives. [file 1745-6215-10-105-S2.PDF]

Dr. Brian Schwartz, Medical Director

TOPHR-HIT INFUSION PROTOCOL **(Revised January 2005)**
(≥ 16 y/o)

When the following conditions exist, an Advanced Care (LIII) Paramedic may initiate the TOPHR-HIT fluid infusion under the following protocol:

CONDITIONS

- Blunt trauma mechanism of injury

AND
- GCS ≤ 8

AND
- Age ≥ 16

EXCLUSIONS

- Known/suspected pregnancy
- Primary penetrating injury
- VSA prior to randomization
- Time interval between arrival at scene and intravenous access exceeds 4 hours
- Amputation above wrist or ankle
- Any burn (thermal, chemical, electrical, radiation)
- Suspected hypothermia
- Asphyxia (strangulation, hanging, choking, suffocation, drowning)
- Fall from height ≤ 1m or ≤ 5 stairs

PROCEDURE

1. Ensure secure airway and spinal precautions as appropriate.
2. Initiate rapid transport as per FTT guidelines.
3. Establish a patent intravenous line, 0.9% Normal Saline, TKVO.
4. Prepare randomized study IV bag with secondary med set.
5. "Piggyback" secondary set into primary IV line.
6. Stop primary IV solution and infuse entire study solution (250 mL) as a rapid bolus.
7. Upon completion of study infusion, close clamp on secondary med set, return 0.9% Normal Saline to TKVO.
8. Assess vital signs and follow Medical Directive: Fluid Therapy Protocol

NOTE
- Patients should be transported as per Field Trauma Triage guidelines to the appropriate trauma centre.
- It is acceptable to start the study infusion as your first IV access
- The fluid administered is 7.5% hypertonic saline in 6% Dextran 70 solution or placebo (0.9% Normal Saline)
