# Supplementary material for: The Toronto prehospital hypertonic resuscitation-head injury and multi organ dysfunction trial (TOPHR HIT) - Methods and data collection tools
Source: Trials. 2009 Nov 20;10:105. doi: 10.1186/1745-6215-10-105 (PMC2788534; doi:10.1186/1745-6215-10-105)
Supplement: Additional file 3 — Blood laboratory Method. [file 1745-6215-10-105-S3.DOC]

**Appendix 3. Blood laboratory Method**

**MATERIALS AND METHODS**

**MONOCLONAL ANTIBODIES AND REAGENTS**

Blood samples were collected in either sodium heparin or K2EDTA BD Vacutainer

venous blood collection tubes as indicated (BD Biosciences; Mississauga, ON). The

flourescent antibody Annexin V-PE as well as those against the surface epitopes CD62LFITC,

CDllb-PE, CD66b-FITC, CD63-PE, CD95-FITC, CD45-PerCP and CDl4-APC

were all obtained from BD Biosciences (Mississauga, ON). The vital dye 7-AAD, was

obtained from BD Biosciences (Mississauga, ON). Anti-CD95L (CDI78)-PE was

acquired from Groovy Blue Genes Biotech Ltd. (Vineland, ON). Annexin V Binding

Buffer (IOmM HepeslNaOH, pH 7.4, 140mM NaC!, 2.5mM CaCb), FACS Lysing

Solution «15% formaldehyde and <50% diethylene glycol), and the active Caspase-3

Antibody Apoptosis kit (containing Cytofix/Cytoperm Solution, PermlWash Buffer and

Caspase-3-PE) were purchased from BD Biosciences (Mississauga, ON).

**BLOOD SAMPLES**

This study received approval from the Sunnybrook and Women's Research Ethics Board

as well as the DRDC Human Research Ethics Committee. Blood samples were drawn at

one timepoint from healthy volunteers at rest and were tested immediately (n=25).

Patient blood samples were taken at 4 timepoints: Emergency Department Admission

(ED Adm), 12h, 24h and 48h after resuscitation. Blood samples were sent to DRDCToronto

immediately for testing.

**WHOLE BLOOD HAEMATOLOGY**

Whole blood samples were collected into K1EDTA Vacutainer tubes and cell counts

taken using the Beckman Coulter Ac·T dift2 Hematology Analyzer. Counts were

completed within 3h ofsample collection.

**SURFACE MARKERS (CD62L, CDllb, CD95, CD95L)**

Adhesion (CD62L-FITC, CDllb-PE), degranulation (CD66b-FITC, CD63-PE) or

apoptotic (CD95-FITC, CD95L-PE) surface markers added in conjunction with CD45PerCP

(for neutrophil selection) were added in saturating concentrations to 100f.lL whole

blood aliquots and incubated 30 minutes in the dark at room temperature, followed by

incubation with IX FACSLysing Solution for 15 minutes in the dark at room

temperature. Samples were centrifuged, supernatant aspirated, and washed with Cell

Wash Buffer (0.1% NaN) and I%BSA in PBS. Each sample was resuspended in 200uL

of I% Formaldehyde in PBS and wash buffer added as needed to dilute further. Results

were acquired by flow cytometry.

**CASPASE-3 ASSAY**

Anti-CD45-PerCP antibody was added to 100f.lL of blood, vortexed and incubated for

15min at room temperature in the dark. FACSLysing (IX) solution was added, followed

by vortexing and incubation 10-15min at room temperature in the dark. After

centrifugation and aspiration of supernatant, blood was washed with cold PBS.

Cytofix/Cytoperm Solution (IX) was added as directed by the Active Caspase-3
