# Supplementary material for: The Toronto prehospital hypertonic resuscitation-head injury and multi organ dysfunction trial (TOPHR HIT) - Methods and data collection tools
Source: Trials. 2009 Nov 20;10:105. doi: 10.1186/1745-6215-10-105 (PMC2788534; doi:10.1186/1745-6215-10-105)
Supplement: Additional file 4 — MRI acquisition parameters. [file 1745-6215-10-105-S4.PDF]

Appendix 4: Structural Magnetic Resonance Imaging (MRI) ACQUISITION PARAMETERS

	Scout	Axial T2 (Spin Echo)	Axial 3D SPGR	Diffusion DW-EPI	FLAIR T2 FLAIR	Gradient ECHO 2D GRE
Imaging Parameters	Sagittal<br>2D	FC<br>VEMP<br>VB (interleave)	Ax Volume<br>SAT (S_1)<br>SPGR	Axial<br>2D	Axial<br>2D	2D<br>axial
Pulse Timing (msec)						
TE	8	30/80	4-2	74.7	140	35
TR	450-50	3000	35	8200	9000	800
Flip Angle (°)	90	90	35	90	90	20
Scanning Range	22 cm FOV<br>5/2.5 mm<br>17-19 slices	20 cm FOV<br>3/0 mm<br>62 slices	22 cm FOV<br>1.2/0 mm<br>128 slices	29 cm FOV<br>5/0 mm<br>27 slices	20-22 cm FOV<br>5/2.5 mm<br>19-24 slices	20 cm FOV<br>6/2 mm<br>18 slices
Acquisition	256x192	256x92<br>.5 Nex	256x192<br>1 Nex	128x192<br>1 Nex	256x192<br>1 Nex	256x192<br>2 Nex
Approximate Scan Time (mins)	3.20	11.36	10.48	0.33	3.36	5.10
