# Supplementary material for: The Toronto prehospital hypertonic resuscitation-head injury and multi organ dysfunction trial (TOPHR HIT) - Methods and data collection tools
Source: Trials. 2009 Nov 20;10:105. doi: 10.1186/1745-6215-10-105 (PMC2788534; doi:10.1186/1745-6215-10-105)
Supplement: Additional file 5 — MRI Scan Parameters. [file 1745-6215-10-105-S5.DOC]

# Appendix 5: Magnetic Resonance Spectroscopy (MRS) SCAN PARAMETERS

| Protocol | PROBE-SV | PROBE–SV |
| --- | --- | --- |
| Sequence | PROBE-P | PROBE-P |
| TR (ms) | 1500 | 1500 |
| TE (ms) | 135 | 30 |
| FOV (cm) | 24 | 24 |
| Voxel Thickness (mm) | 20 | 20 |
| VOI (ml) | 8 | 8 |
| Echoes | 1 | 1 |
| Scans | 128 | 128 |
| NEX | 8 | 8 |
| Number of VOIs | 4 | 4 |
| Scan Time (hh:mm:ss) | 00:24:33 | 00:24:33 |
