# Supplementary material for: The Toronto prehospital hypertonic resuscitation-head injury and multi organ dysfunction trial (TOPHR HIT) - Methods and data collection tools
Source: Trials. 2009 Nov 20;10:105. doi: 10.1186/1745-6215-10-105 (PMC2788534; doi:10.1186/1745-6215-10-105)
Supplement: Additional file 6 — MRI brain atrophy. [file 1745-6215-10-105-S6.PDF]

## APPENDIX 6: BRAIN REGIONAL ATROPHY AND HYPERINTENSITY MEASUREMENT PROTOCOL

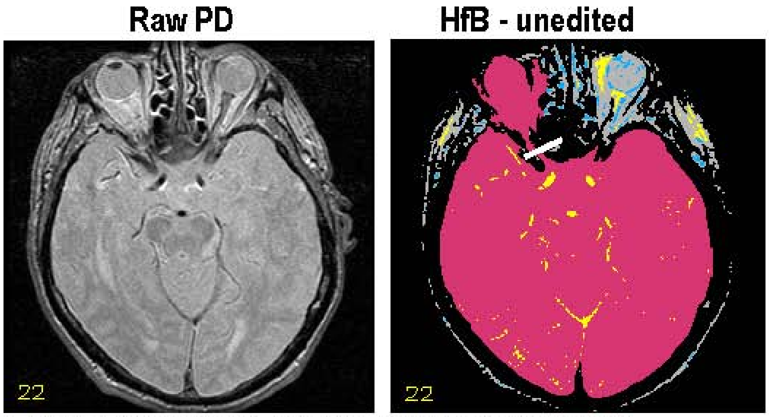


**1.a. Skull Removal** - Skull is removed using semi-automatic procedure based on two-dimensional PD/T2 histogram

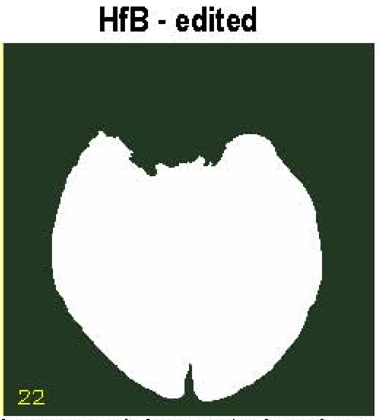


**1.b.** Binary mask is created and coregistered into T1 space, as T1 segmentation requires skull removed

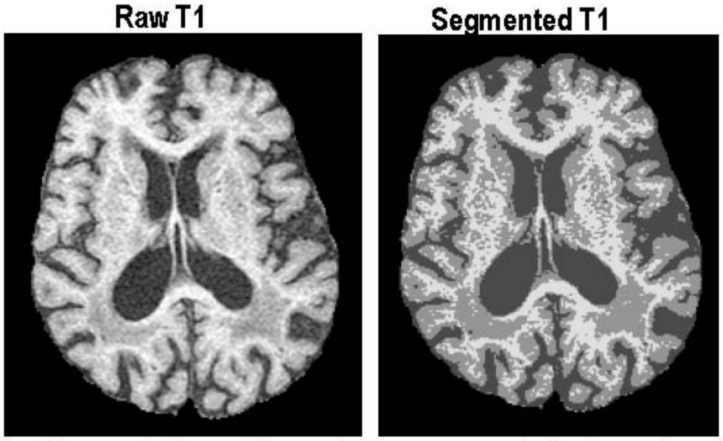


**2.a. Segmentation** - T1 scan is then segmented using automatic process which accounts for scan inhomogeneneity. Algorithm fits gaussian curves to T1 histograms.

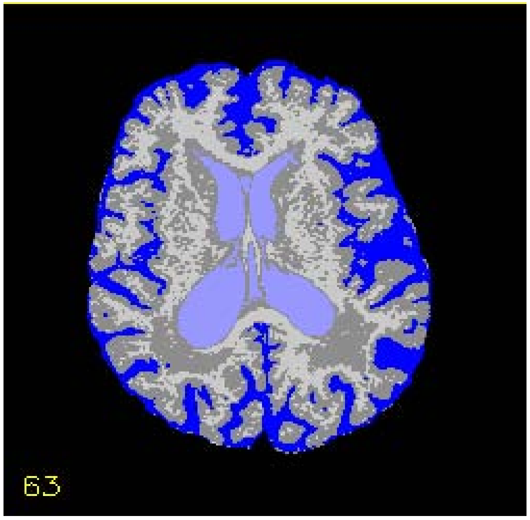


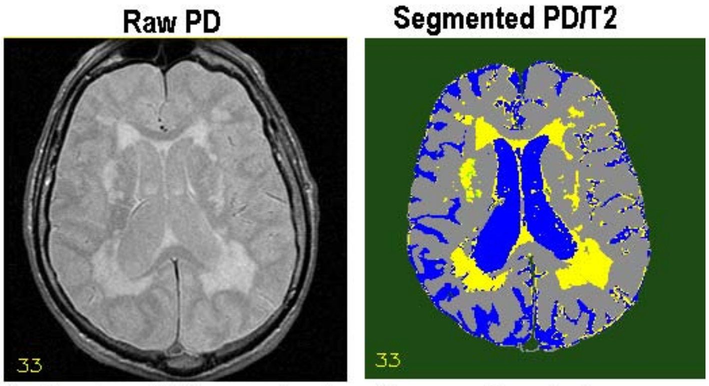


**3.a. Lesion** - Using semi-automatic procedure, lesions are localized based on 2D histogram of PD/T2.

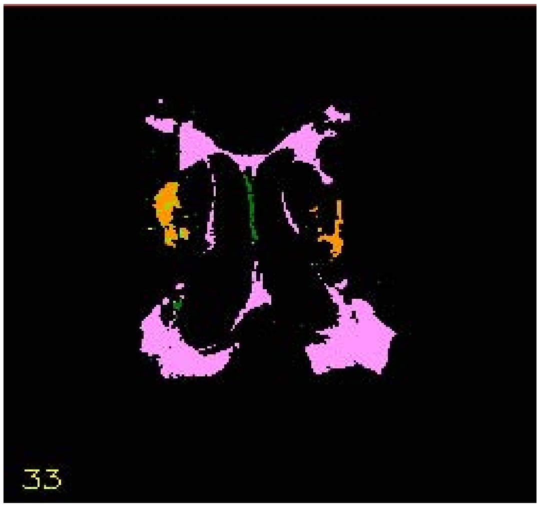

4.**Tri-feature Coregistration**-T1 and PD/T2 scans are aligned into AC-PC space. Grey matter, white matter and CSF information are taken from T1. Lesion information comes from PD/T2. This information is combined to produce global counts.

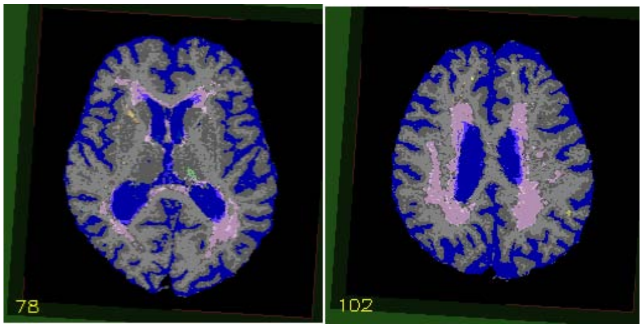


5.a. **Regional Parcellation-Surface Rendered View** -Brain is AC-PC aligned, and divided into sections based on sulcallandmarks and stereotaxicTalairachgrid.

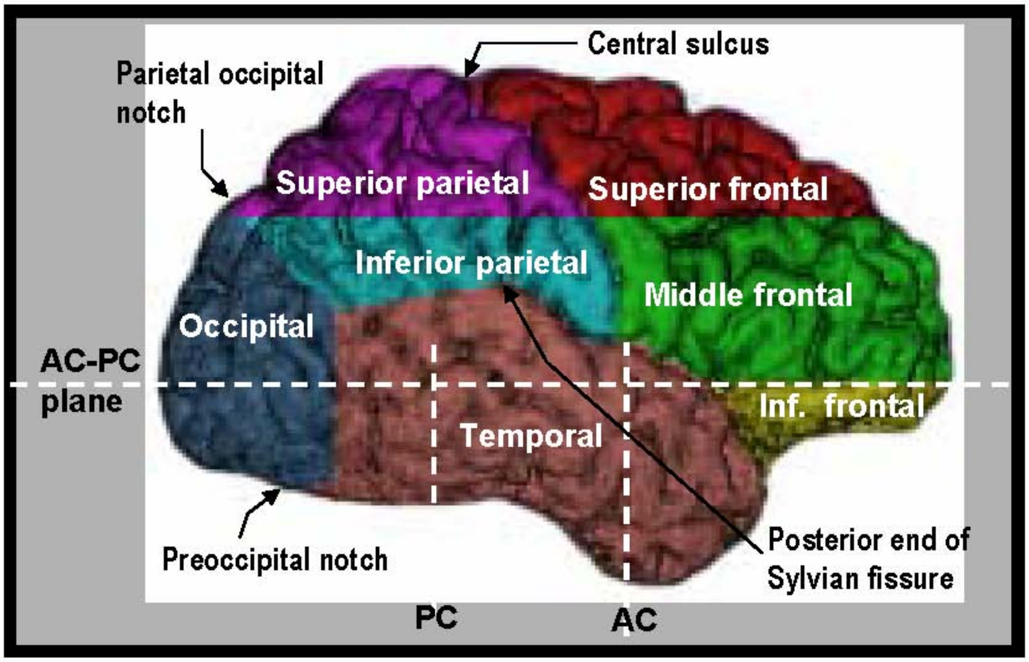


**5.b. Axial View** - When lobar mask is applied to the combined image, information regarding regional tissue and lesion volumes is obtained.

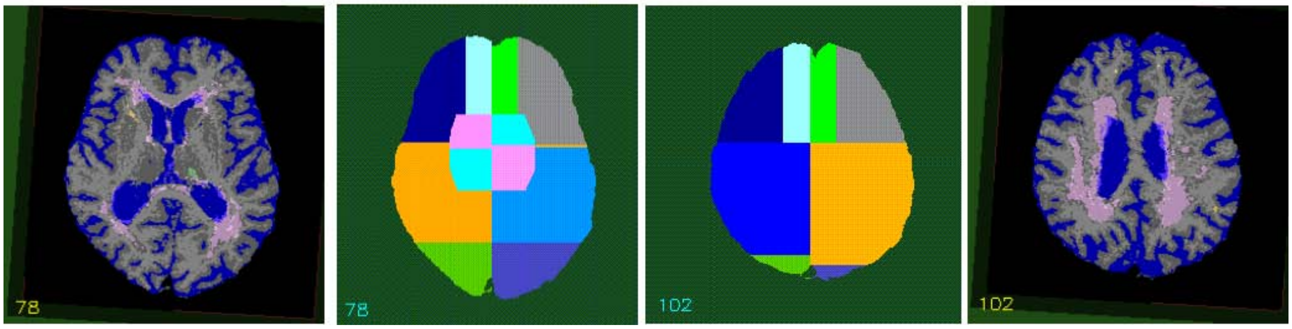


*Sunnybrook & Women's College HSC / RotmanResearch Institute, BaycrestCentre for Geriatric Care, University of Toronto*
