# Supplementary material for: The Toronto prehospital hypertonic resuscitation-head injury and multi organ dysfunction trial (TOPHR HIT) - Methods and data collection tools
Source: Trials. 2009 Nov 20;10:105. doi: 10.1186/1745-6215-10-105 (PMC2788534; doi:10.1186/1745-6215-10-105)
Supplement: Additional file 7 — MRI DTI. [file 1745-6215-10-105-S7.DOC]

# Appendix 7: Diffusion Tensor Imagery (DTI) SCAN PARAMETERS

| **Imaging Parameters** | **Patient Position** | supine |
| --- | --- | --- |
|  | **Patient Entry** | head first |
|  | **Coil** | head coil |
|  | **Plane** | oblique |
|  | **Mode** | 2D |
|  | **Pulse Sequence** | SE |
|  | **Imaging Options** | none |
|  | **Psd Name** | /research/dti/depi |
| **Pulse Timing** | **# of Shots** | 1 |
|  | **TE** | minimum |
|  | **TR** | 3000 |
|  | **bandwidth** | 62.50 |
| **Scanning Range** | **FOV** | 24 cm |
|  | **slice thickness** | 4 mm |
|  | **spacing** | 0 mm |
|  | **Number of slices** | 8 |
| **Acquisition** | **frequency** | 128 |
|  | **phase** | 128 |
|  | **NEX** | 1 |
|  | **Phase FOV** | 1 |
|  | **Freq Direction** | R/L |
|  | **Auto Center Freq** | water |
| **User CV’s** | **Bo** | 500 |
|  | **# gradient directions** | 10 |
| Approximate scan time |  | 4 min |
