# Supplementary material for: The Toronto prehospital hypertonic resuscitation-head injury and multi organ dysfunction trial (TOPHR HIT) - Methods and data collection tools
Source: Trials. 2009 Nov 20;10:105. doi: 10.1186/1745-6215-10-105 (PMC2788534; doi:10.1186/1745-6215-10-105)
Supplement: Additional file 8 — MRI MTLT in AD. [file 1745-6215-10-105-S8.PDF]

Appendix 8

Thinnest MTL Thickness(MTLT) in Alzheimer's Disease (AD)

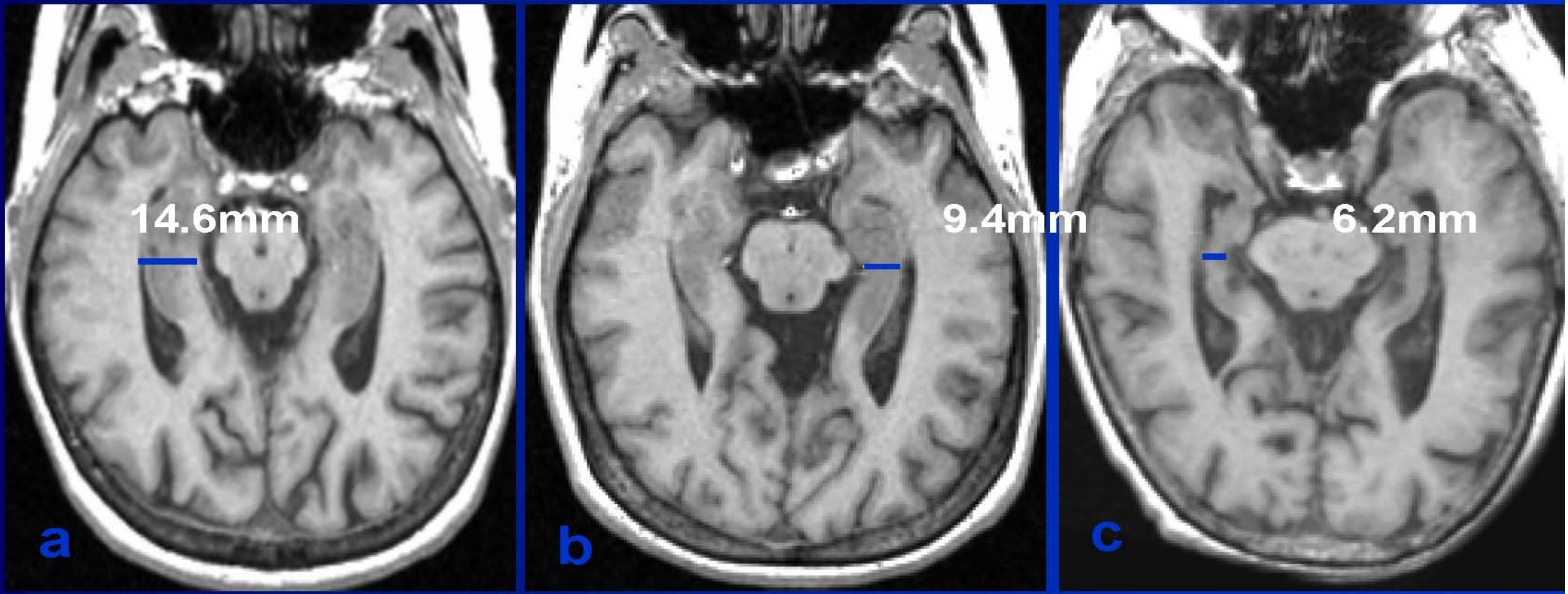

NC — Mild AD — Moderate AD

Sens: 86%

Spec: 95%. Accuracy: 92%

Gao et al., *Neurobiol.Aging (in p*
