# Supplementary material for: The Toronto prehospital hypertonic resuscitation-head injury and multi organ dysfunction trial (TOPHR HIT) - Methods and data collection tools
Source: Trials. 2009 Nov 20;10:105. doi: 10.1186/1745-6215-10-105 (PMC2788534; doi:10.1186/1745-6215-10-105)
Supplement: Additional file 9 — MRI DTI in AD. [file 1745-6215-10-105-S9.PDF]

Appendix 9: ILLUSTRATING DIFFUSION TENSOR IMAGING IN A PATIENT WITH PROBABLE ALZHEIMER'S DISEASE

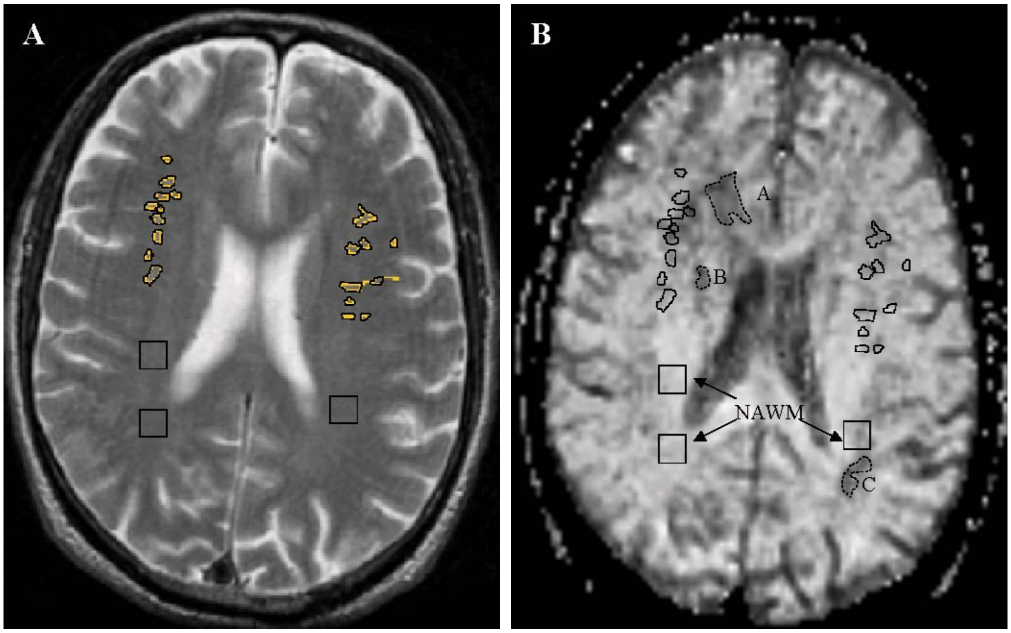

	Mean Fractional anisotropy +/- standard dev	
	right	left
**WMSH**	**0.59 +/- 0.08**	**0.67 +/- 0.07**
**Region A**	**0.44 +/- 0.10**	
**Region B**	**0.45 +/- 0.12**	
**Region C**		**0.59 +/- 0.13**
**NAWM**	**0.728 +/- 0.072 (mean of 3 regions)**	
