# Supplementary material for: The Toronto prehospital hypertonic resuscitation-head injury and multi organ dysfunction trial (TOPHR HIT) - Methods and data collection tools
Source: Trials. 2009 Nov 20;10:105. doi: 10.1186/1745-6215-10-105 (PMC2788534; doi:10.1186/1745-6215-10-105)
Supplement: Additional file 10 — Paramedic data checklist. [file 1745-6215-10-105-S10.PDF]

TOPHR HIT PARAMEDIC DATA CHECKLIST

If lost please return to the Prehospital and Transport Medicine Research Program, Tel: 416-480-5339

To secure your incentive, please ensure that you fill out each section completely and accurately. All information is confidential and only for research use.

Run Number: ____________________ Patient Health Insurance Number (HIN): ___________________

Randomization Number: ____________________ Did you remember to label the infusion bags? □ Yes □ No

Please check mark if patient met the Inclusion & Exclusion Criteria:

Inclusion Criteria: □ Age ≥ 16 □ Blunt trauma mechanism of injury □ GCS ≤ 8 □ Trauma centre: SMH or S & W
□ IV Access

Exclusion Criteria:
- □ No known or suspected pregnancy
- □ No VSA prior to randomization
- □ Arrive scene to IV access is not >4 hours
- □ No burn (thermal, chemical, electrical, radiation)
- □ No asphyxia (strangulation, hanging, choking, suffocation, drowning)
- □ No primary penetrating injury
- □ Fall from height is not ≤ 1m or ≤ 5 stairs
- □ No amputation above wrist or ankle
- □ No suspected hypothermia (severe < 32°C)

Basics of Mechanism: □ Transport (vehicle or pedestrian) □ Fall □ Interpersonal violence
□ Other, please specify: ________________

Date Trauma Occurred: __ __ __ __ / __ __ / __ __ (y y y y m m d d) Time Trauma Occurred: __ __ / __ __ (h h m m)

Date Trauma Recognized: __ __ __ __ / __ __ / __ __ (y y y y m m d d) Time Trauma Recognized: __ __ / __ __ (h h m m)

Trauma Witnessed: □ Yes □ No

If yes, Trauma Witnessed By: □ Toronto EMS □ Layperson bystander(s) □ Off duty medical professional(s)
□ Other, please specify: ____________________

Bystander Care: □ Yes □ No

If yes, Bystander Care Provided By: □ Layperson bystander(s) □ Off duty medical professional(s)
□ Other, please specify: ____________________

Patient intubated prior to study fluid administration: □ Yes □ No

Please check mark each box below to ensure you are the crew that performed the following:

□ Administered the study fluid □ Transported the patient □ Completed the ACR

If you answered yes to all three tasks above, please indicate the following:

Attendant Name: __________________ Oasis Number: ____________________ Incentive Choice: __________________
(*Home Depot, Mountain Equ., Cadillac Fairview, Indigo*)

Driver Name: __________________ Oasis Number: ____________________ Incentive Choice: __________________
(*Home Depot, Mountain Equ., Cadillac Fairview, Indigo*)

Student Name: __________________ Oasis Number: ____________________ Incentive Choice: __________________
(*Home Depot, Mountain Equ., Cadillac Fairview, Indigo*)

Other Name: __________________ Oasis Number: ____________________ Incentive Choice: __________________
(*Home Depot, Mountain Equ., Cadillac Fairview, Indigo*)

Vehicle Number: __________________ Originating Station Number: ____________________

For other crews on scene, please indicate the following:

1) Vehicle Number: __________ Station Number: __________ ACP or PCP: __________

2) Vehicle Number: __________ Station Number: __________ ACP or PCP: __________

3) Vehicle Number: __________ Station Number: __________ ACP or PCP: __________

Patient received the full amount (250 mL) of the study fluid: □ Yes □ No

Next page

TOPHR HIT PARAMEDIC DATA CHECKLIST

If lost please return to the Prehospital and Transport Medicine Research Program, Tel: 416-480-5339

If yes, Date Infusion Completed: ___ ___ ___ ___ / ___ ___ / ___ ___ (y y y y m m d d)   Time Infusion Completed: ___ ___ / ___ ___ (h h m m)

If no, Total Amount Infused: ____________________mL

Reason for Incomplete Infusion of Study Fluid:

□ IV stopped due to poor connection
□ IV stopped due to complication/adverse event, please specify: ____________________
□ IV pulled out by patient
□ IV interstitial
□ IV stopped by hospital staff
□ Other, please specify: ____________________

Patient Received: □ **Hypertonic Saline 7.5% Dextran 6% (250 ml)** □ **Sodium Chloride 0.9% (250 ml)**

Was the trauma team notified of the study fluid the patient received: □ Yes □ No
(FYI…the trauma team should not have been notified of solution type; only that the patient was enrolled into this trial)

Study fluid is easy to administer: 1 2 3 4 5 (Agree … Disagree)   Medical directive is easy to follow: 1 2 3 4 5 (Agree … Disagree)

Do you have any hesitation to using HSD in the field? □ Yes □ No □ Other, please specify: ____________________

If yes, please indicate why: ____

CTAS Score (depart scene):
□ 1 Resuscitation
□ 2 Emergency
□ 3 Urgent
□ 4 Less urgent
□ 5 Non urgent
□ 6 Not applicable

CTAS Score (arrive hospital):
□ 1 Resuscitation
□ 2 Emergency
□ 3 Urgent
□ 4 Less urgent
□ 5 Non urgent
□ 6 Not applicable

Patient Status Change Score:
□ 1 Improved
□ 2 Unchanged
□ 3 Worsened
□ 4 Became VSA en route
□ 5 Not applicable

Case Severity Score:
□ 1 Minor
□ 2 Moderate
□ 3 Severe
□ 4 Life threatening
□ 5 VSA

Did you deviate from the TOPHR HIT medical directive? □ No □ Yes

If yes, please indicate why: ____

Please ensure the following critical data points and times are documented on the ACR (collected prior to treatment, post treatment and arrival to ED):

□ Pulse	□ Pupils	□ Temperature	□ End Tidal CO_2
□ ECG code	□ Immobilization	□ Capillary glucose	□ Respirations
□ Airway management	□ Sat O_2	□ Oxygen therapy	□ IV fluids
□ GCS (E, V, M and total)	□ Blood pressure	□ Ventilation	

Further, please ensure the following are also documented on the ACR:

□ Health Insurance Number	□ Depart scene	□ Postal code	□ Medical History
□ Arrival Patient	□ Patient address	□ Station number	□ Weight
□ Date of Birth	□ Vehicle number	□ Crew mobile	□ Arrival Scene
□ Gender	□ Breathing complications	□ Surname	□ Given name
□ Medications	□ Call time	□ Age	
□ Call Date	□ Arrival hospital	□ IV attempts	

Attendant please sign and date:

Print Name: __________________________ Signature: ________________________ Date: ____________________ (mmm/dd/yyyy)
