# Supplementary material for: The Toronto prehospital hypertonic resuscitation-head injury and multi organ dysfunction trial (TOPHR HIT) - Methods and data collection tools
Source: Trials. 2009 Nov 20;10:105. doi: 10.1186/1745-6215-10-105 (PMC2788534; doi:10.1186/1745-6215-10-105)
Supplement: Additional file 11 — Pre-hospital CRF-LAND. [file 1745-6215-10-105-S11.PDF]

THE TORONTO PREHOSPITAL HYPERTONIC RESUSCITATION - HEAD INJURY AND MULTI ORGAN DYSFUNCTION FEASIBILITY TRIAL (TOPHR HIT) PREHOSPITAL LAND VEHICLE CASE REPORT FORM (FORM 1/4)

Rules: *Fill fields from left to right. For fields where no information is available, use a yellow highlighter to mark the entire field; this signifies that the field was intentionally left blank because no information was available for abstraction.*

Tyrone Perreira, B.PHE, ACP, Paramedic Research Coordinator, Prehospital and Transport Medicine Research Program, SWCHSC Tel: 416-480-6100x7072 Email: tyrone.perreira@sw.ca

STUDY PATIENT NUMBER & CALL INFORMATION

v1 **Subject ID Code** *(Assigned by Paramedic Research Coordinator)* _____ _____ _____

v2a **Air or Land Vehicle** *(For LAND cases an "L" should be entered into the dataset; for AIR cases use an "A""; for CRITICAL CARE Cases use a "C".)* L

v2b **Patient Number** *(EMS patient number as entered into the "Administration" section–"Call Number/Patient Number" field from the ACR that was completed by the ACP crew that administered the study fluid and transported the patient (i.e., **treating ACP crew (TACPC)**). The ACR call information must be identical to the TOPHR HIT Data Checklist call information (e.g., Call Number))* ___ ___ ___ ___ ___ ___ ___ ___ ___ ___ ___

v3, v4 **Call Received Date** *(TACPC ACR Administration - Call Date (YMD) **Convert 2 digit month to 3 character month**)*

____ ____ ____ ____ / ____ ____ ____ / ____ ____
y y y y m m m d d

Call Received Time *(transcribe from the CAD that matches the TACPC ACR and **not** from ACR General Administration – Call Received (since it's less accurate). The relevant CAD is determined by: matching location or vicinity, then date and times to the TACPC ACR)*

___ ___ : ___ ___ : ___ ___ (00:00:00 (midnight) – 23:59:59)
h h m m s s

v5 **Multiple CADs** *(if different call numbers are available for the same location or vicinity with similar date/times. Confirm whether they represent the same incident)*

1 = Yes / **2** = No / **3** = Unknown

↓ **If *Yes*,**

v6 **Earliest Call Received Date** *(if multiple calls were received for the same incident and multiple CAD sheets are available, then transcribe the earliest call received date and time from the earliest CAD. Confirm date that spans midnight).*

____ ____ ____ ____ / ____ ____ ____ / ____ ____
y y y y m m m d d

v7 **Earliest Call Received Time**

___ ___ : ___ ___ : ___ ___ (00:00:00 (midnight) – 23:59:59)
h h m m s s

v8 **Vehicle Type** *(TACPC ACR - top right page 1 - Crew Type)* **1**=PCP **2**=ACP **3**=CCP

v9 **Vehicle Number** *(TACPC ACR Administration – Vehicle Number, transcribe as is)* ____ ____ ____ ____

v10 **Station** *(TACPC ACR Administration – Station: transcribe as is in first two spaces of field; Paramedic code: transcribe as is into third space of field)* ____ ____ ____ (01 – 49, 0=EMA, 7=PCP, 8=ACP, 6=CC, 9=P)

v11 **Vehicle Status** *(TACPC ACR Administration – Status)* ____ ____ (00=At Base, 77=Mobile, 88=Standby loc., 99=Maintenance)

v12 **Crew Member 1 (Attendant) No.** *(TACPC OASIS # from ACR General Administration – Crew Member 1 (Attendant) No.)* ____ ____ ____ ____ ____ ____

INCLUSION CRITERIA

v13	**Age ≥16 years** *((TACPC ACR Administration – Date of Birth (YMD) or estimated age from ACR – Physical Exam – Age (must be ≥ 16 years) and* ***TOPHR HIT Paramedic Data Checklist (TPDC)*** *– Inclusion Criteria – Age ≥ 16. All form discrepancies must be resolved prior to Study ID Code assignment)*	**1** = Yes / **2** = No <STOP>
v14	**Blunt Traumatic Injury** *((TACPC ACR –Clinical Information – Traumatic Injury Site/Type - Type column=34 or 35 or Incident History or General Information-Remarks) and TPDC–Inclusion Criteria –Blunt Trauma. Note "crush injury" is considered to be a form of blunt trauma here)*	**1** = Yes / **2** = No <STOP>
v15	**GCS** *(total GCS score prior to study fluid from TACPC ACR – Clinical Information – Incident History; Clinical Treatment/Procedures & Results (**CTPR**) and TPDC – Inclusion Criteria - GCS≤8. To be included total score must range from ≥3 - ≤8)*	**1** = Yes / **2** = No <STOP>
v16	**ACP Treated, Transported, and ACR** *(one ACP crew administered the study IV fluid and the same, or another, ACP crew transported the patient (if the patient was not pronounced at scene) and completed the ACR, which was used for this CRF as the original source document (ACR yellow copy). Aka TACPC for "treating ACP crew". And the TPDC - TACPC treated, transported, and ACR boxes are checked. Level I or II precepting as Level III (with a preceptor) are included. Helicopter transport cases are decided via consensus)*	**1** = Yes / **2** = No <STOP>
v17	**IV Access** (*IV access obtained at any point during the call TACPC ACR – CTPR - IV Procedures Codes or text (e.g., "IV running well" or "IV TKVO") and TPDC – IV Access. Do not use top right Call Type "☐ IV ONLY" since call limited to only IV given. IV codes include 340 and 345 (Normal Saline) If patient entered in the trauma room, circle 'yes.')*	**1** = Yes / **2** = No <STOP>

EXCLUSION CRITERIA

v18	**Known or Suspected Pregnancy** *(TPDC – Exclusion Criteria–No Known or suspected pregnancy. Not using TACPC ACR unless necessary since no pertinent negatives are systematically recorded on ACR)*	**1** = Yes <STOP> / **2** = No
v19	**Primary Injury Penetrating** *(TPDC – Exclusion Criteria – No primary penetrating trauma)*	**1** = Yes <STOP> / **2** = No
v20	**VSA Prior to Randomization** *(TPDC – Exclusion Criteria – No VSA prior to randomization)*	**1** = Yes <STOP> / **2** = No
v22	**Arrival at scene to IV access >4 hours** *(TACPC ACR CTPR – 1st IV access time minus TACPC ACR General Administration – Arrive Scene is less than or equal to 4 hours and TPDC – Exclusion Criteria – No time to IV access > 4 hours)*	**1** = Yes <STOP> / **2** = No
v23	**Amputation above wrist or ankle** *(TPDC – Exclusion Criteria –No amputation above wrist or ankle)*	**1** = Yes <STOP> / **2** = No
v24	**Any burn** *(TPDC – Exclusion Criteria – No burn (thermal, chemical, electrical, radiation))*	**1** = Yes <STOP> / **2** = No
v25	**Suspected hypothermia** *(TPDC – Exclusion Criteria – No suspected hypothermia)*	**1** = Yes <STOP> / **2** = No
v26	**Asphyxia** *(TPDC – Exclusion Criteria – No asphyxia)*	**1** = Yes <STOP> / **2** = No
v27	**Minor Fall** *(no minor fall, i.e., no fall from standing on floor, no fall from height ≤ 1 m, or no fall from ≤ 5 stairs (TACPC ACR or TPDC))*	**1** = Yes <STOP> / **2** = No

PATIENT DEMOGRAPHICS

v28 **Date of birth** *(TACPC ACR Administration – Date of Birth (YMD). Must be ≥ 16 years)*

____ ____ ____ ____ / ____ ____ ____ / ____ ____
y y y y m m m d d

v29 **Estimated Age** *(transcribe as recorded on TACPC ACR – Physical Exam – Age, e.g., "60ish" is transcribed as "60ish". Must be ≥ 16 years)*

__ __ __ __ __ __ (016 years – 110 years)

V30 **Gender** *(TACPC ACR – Physical Exam - Gender)*

(**M**)ale / (**F**)emale / (**U**)known / (**N**)ot available

v31 **Estimated Weight** *(TACPC ACR – Physical Exam – Weight (kg). If recorded in pounds, Paramedic Research Coordinator to enter the equivalent in kilograms (kgs = pounds/2.2). Add leading zero)*

_____ _____ _____ kg (approx. 040 kg – 250 kg)

CIRCUMSTANCES OF INJURY

v32 **Type of Injury** *(TACPC ACR – Clinical Information – Traumatic Injury Site/Type - Type column = 34 or 35 or Incident History or General Administration - Remarks / Orders)*

1. Blunt (34) / **2**. Crush (35) / **3**. Both / **4**. Other, specify:

v33 **Case Severity Score** *(this is the paramedic impression of case severity and is determined at call completion. Among other things, case severity determines "Warning Signals". Though this field is not available from the new ACR, paramedics are still asked to record this on the Checklist - see TPDC – Case Severity Score. The highest severity score is transcribed to the TPDC)*

1. Minor
2. Moderate
3. Severe
4. Life Threatening
5. VSA
6. Missing

v34 **Mechanism of Injury** *(TACPC ACR –Clinical Information – Traumatic Injury Site/Type – Mechanism column and TPDC – Basics of Mechanism)*

1.Transport / **2**.Fall / **3**.Interpersonal Violence / **4**.Other, specify: ____
58,59,60,61 / 53,54 / 50,55,63 / 52,57,62,64,65

↓ **If *Transport,***

v35 **Type of Transport** (If *Transport*, then please specify type of transport)

1. MVC (car or truck (58))
2. MOTORCYCLE/RECREATIONAL VEHICLE (59)
3. PEDAL BICYCLE (60)
4. PEDESTRIAN STRUCK (61)
5. Missing
6. Other, please specify:

v36 **Deliberate Self-Harm** *(TACPC ACR Clinical Information – Incident History)*

1=Yes / **2**=Suspected or possible / **3**=No mention on ACR / **4**=No

PREHOSPITAL SYSTEM FACTORS

v37, v38

Trauma Occurs Date *(TPDC – Trauma Occurred Date. This date/time may or may not be the same as the trauma recognized date/time and/or call received date/time. If missing, see TACPC – ACR – Clinical Information – Incident History then decide via consensus, create a note-to-file to document, and enter the estimated date (e.g., "incident occurred 1 hour previous" and arrive scene=02Jan2005 00:30 hours then trauma occurs date=01Jan2005 23:30 hours. Confirm date that spans midnight).*

____ ____ ____ ____ / ____ ____ ____ / ____ ____
y y y y m m m d d

Trauma Occurs Time *(TPDC – Trauma Occurs Time. If missing, and relevant information is available from the TACPC ACR (e.g., "trauma occurred 20 minutes ago") then decide via consensus, create a note-to-file to document, and enter the estimated time. Note: cannot use ACR – Clin information – "Time of Occurrence" since the definition for "occurrence" is not standard)*

___ ___ : ___ ___ (00:00 (midnight) – 23:59)
h h m m

v39, v40 **Trauma Recognized Date**
(date trauma was recognized or "discovered" or "witnessed" by bystander or EMS. TPDC – Trauma Recognized Date. If missing, see TACPC – ACR – Clinical Information – Incident History and decide via consensus (see above). Confirm date that spans midnight).

___ ___ ___ ___ / ___ ___ ___ / ___ ___
y y y y m m m d d

Trauma Recognized Time
(TPDC – Trauma Recognized Time)

___ ___ : ___ ___ (00:00 (midnight) – 23:59)
h h m m

v41 **Trauma Witnessed** *(TPDC – Trauma Witnessed (the trauma or "incident" was witnessed by layperson bystander(s), medical professional(s), or EMS personnel) or TACPC – ACR – Clinical Info Incident History or General Administration - Remarks / Orders)*

1 = Yes / **2** = Not documented on TPDC or ACR / **3** = No

↓ ***If Yes,***

v42 *If EMS witnessed as well as other witness(es) then go with EMS Witnessed. ACR only records "Arrest Witnessed By".*

Type of Witness *(If Witnessed, then please specify type of witness. TPDC – Type of Witness. If missing see TACPC – ACR – Clinical Information – Incident History*

1. Layperson bystander(s)
2. Toronto EMS
3. Off duty medical professional(s) – Utstein
4. Other, please specify:

V43 **Bystander Present** *(TACPC – ACR notes bystander present regardless of bystander witnessed or not or bystander care)*

1. No
2. Yes, layperson(s) present
3. Not Documented on ACR
4. Other: please specify:

↓ **If *Yes or Other,***

v44 **Bystander Care** *(TPDC; Basic Trauma Life Support (BTLS) prior to TACPC arrival to scene by bystander regardless of witnessed or not TACPC – ACR – Clinical Information – Incident History or Treatment prior to arrival or General Administration - Remarks / Orders)*

1. No
2. Yes, layperson(s) provided care
3. Yes, off duty medical professional provided care (i.e., doctor, nurse, or technician) – Utstein
4. Not Documented
5. Other: please specify:

V45 **Other EMS Response**
(mention of other EMS response TACPC arrival at patient. See TPDC or TACPC – ACR – Clinical Information – Incident History or Treatment prior to arrival or CTPR or General Administration – Remarks/Orders)

1 = Yes / **2** = Not documented on ACR / **3** = No

↓ ***If Yes,***

v46	**Fire**	**1** = Yes / **2** = Not documented on ACR / **3** = No
v47	**Police**	**1** = Yes / **2** = Not documented on ACR / **3** = No
v48	**E.F.R.T**	**1** = Yes / **2** = Not documented on ACR / **3** = No
v49	**Other Ambulance**	**1** = Yes / **2** = Not documented on ACR / **3** = No

↓ **If *Yes,***

V50	**ACP 1** *(ACP crew 1 Quad 5-8 & no study fluid given)*	**1** = Yes / **2** = Not documented on ACR / **3** = No
v51	**ACP 2** *(ACP crew 2 Quad 5-8 & no study fluid given)*	**1** = Yes / **2** = Not documented on ACR / **3** = No
v52	**PCP 1** *(PCP crew 1 ACR - Vehicle No. Quadrant 1-4)*	**1** = Yes / **2** = Not documented on ACR / **3** = No
v53	**PCP 2** *(PCP crew 1 ACR - Vehicle No. Quadrant 1-4)*	**1** = Yes / **2** = Not documented on ACR / **3** = No
v54	**Other Crew** *(e.g., 3rd PCP with Preceptor)*	**1** = Yes / **2** = Not documented on ACR / **3** = No<br>↓ **if *Yes,*** pls specify:
v55	**Other**	**1** = Yes / **2** = Not documented on ACR / **3** = No<br>↓ **if *Yes,*** pls specify:

Date/Time Points (if no times are documented, please leave date blank)

v56, v57

Crew Notified Date *(derive date TACPC crew notified from TACPC ACR – Call Date and CTPR and if missing then derive based on ACR – General Administration - Call Events- Crew Notified and confirm date that spans midnight).*

___ ___ ___ ___ / ___ ___ ___ / ___ ___
y y y y m m m d d

Crew Notified Time *(TACPC ACR – General Administration- Call Events- Crew Notified)*

___ ___ : ___ ___ (00:00 (midnight) – 23:59)
h h m m

v58, v59

Vehicle Moves Date *(derive date TACPC vehicle moves or from TACPC ACR – Call Date and CTPR and if missing then derive based on ACR – General Administration- Call Events- Crew Mobile and confirm date that spans midnight).*

___ ___ ___ ___ / ___ ___ ___ / ___ ___
y y y y m m m d d

Vehicle Moves Time *(time TACPC vehicle moves or "crew mobile" from TACPC ACR – General Administration- Call Events- Crew Mobile)*

___ ___ : ___ ___ (00:00 (midnight) – 23:59)
h h m m

V60, v61

Vehicle Stops Date *(derive date TACPC vehicle stops upon arrival at scene TACPC ACR – Call Date and CTPR and if missing then derive based on ACR – General Administration - Call Events – Arrive Scene. Confirm date that spans midnight).*

___ ___ ___ ___ / ___ ___ ___ / ___ ___
y y y y m m m d d

Vehicle Stops Time *(TACPC ACR General Administration – Call Events – Arrive Scene)*

___ ___ : ___ ___ (00:00 (midnight) – 23:59)
h h m m

v62, v63

Arrival at Patient Date *(derive date TACPC is out of vehicle and arrives by patient side TACPC ACR –Call Date and CTPR and if missing only then derive based on ACR – General Administration- Call Events – Arrive Patient. CTPR – any text referencing 'patient contact.' Confirm date that spans midnight).*

___ ___ ___ ___ / ___ ___ ___ / ___ ___
y y y y m m m d d

Arrival at Patient Time *(TACPC ACR General Administration – Call Events – Arrive Patient)*

___ ___ : ___ ___ (00:00 (midnight) – 23:59)
h h m m

v64, v65

Vehicle Departs Scene Date *(derive date TACPC vehicle departs scene with patient TACPC ACR – Call Date and CTPR and if missing then derive based on ACR – General Administration- Call Events – Depart Scene. Confirm date that spans midnight).*

___ ___ ___ ___ / ___ ___ ___ / ___ ___
y y y y m m m d d

Vehicle Departs Scene Time *(TACPC ACR General Administration – Call Events – Depart Scene)*

___ ___ : ___ ___ (00:00 (midnight) – 23:59)
h h m m

v66, v67

Arrival at Facility Date *(derive date TACPC vehicle arrives at SWHSC or SMH with patient TACPC ACR – Call Date and CTPR and if missing then derive based on ACR – General Administration- Call Events – Arrive Dest. Confirm date that spans midnight).*

___ ___ ___ ___ / ___ ___ ___ / ___ ___
y y y y m m m d d

Arrival at Facility Time *(TACPC ACR General Administration – Call Events – Arrive Dest.)*

___ ___ : ___ ___ (00:00 (midnight) – 23:59)
h h m m

PATIENT ASSESSMENT

v67a

Lowest Recorded GCS *(Refer to TACPC ARC – Clinical Information – Incident history or Clinical Treatment / Procedures & Results)* ___ ___ (03 – 08)

v67b

Lowest GCS Assigned **1** = By TACPC / **2** = Prior to Arrival

Vitals Pre/Post Intervention *(closest value prior to and post study fluid transcribed as is from TACPC ACR – Clinical Treatment/Procedures & Results. If pre/post vitals are “staged” across rows, record nearest time. If values are missing from ACR, leave fields blank. When filling out Pre Intervention, please start at v68d-83d and work upwards. If date spans midnight, confirm with CAD. In Diastolic field, use code 999 to represent “P”. In GCS Verbal, use “T” to represent Intubation. For GCS Total, max is either 15(_) or 10(T) depending on if value for GCS Verbal is numeric or “T”.* ***If this is a MISSED CASE (Subject ID Code >=200) “Pre” and “Post” refers to pre and post IV initiation****. Please print clearly.)*

				Pulse	Resp.	Blood Pressure						GCS				Pupils	
		Date yyyy/mmm/dd	Time hh:mm	Rate (mins)	Rate (mins)	Sys. mmHg	Dia. mmHg (P=999)	Temp °C	ECG Code	O_2 Sat %	End T. CO_2 mmHg	E	V	M	Total max 15(_) or 10(T)	R +/-	L +/-
v68-v83	Pre	/ /	:	_ _ _	_ _ _	_ _ _	_ _ _	_ _ ._	_ _	_ _ _	_ _	_	_	_	_ _ (_)	_ _	_ _
v68a - v83a	Pre	/ /	:	_ _ _	_ _ _	_ _ _	_ _ _	_ _ ._	_ _	_ _ _	_ _	_	_	_	_ _ (_)	_ _	_ _
v68b - v83b	Pre	/ /	:	_ _ _	_ _ _	_ _ _	_ _ _	_ _ ._	_ _	_ _ _	_ _	_	_	_	_ _ (_)	_ _	_ _
v68c - v83c	Pre	/ /	:	_ _ _	_ _ _	_ _ _	_ _ _	_ _ ._	_ _	_ _ _	_ _	_	_	_	_ _ (_)	_ _	_ _
v68d - v83d	Pre	/ /	:	_ _ _	_ _ _	_ _ _	_ _ _	_ _ ._	_ _	_ _ _	_ _	_	_	_	_ _ (_)	_ _	_ _
v84 - v99	Post	/ /	:	_ _ _	_ _ _	_ _ _	_ _ _	_ _ ._	_ _	_ _ _	_ _	_	_	_	_ _ (_)	_ _	_ _
v84a - v99a	Post	/ /	:	_ _ _	_ _ _	_ _ _	_ _ _	_ _ ._	_ _	_ _ _	_ _	_	_	_	_ _ (_)	_ _	_ _
v84b - v99b	Post	/ /	:	_ _ _	_ _ _	_ _ _	_ _ _	_ _ ._	_ _	_ _ _	_ _	_	_	_	_ _ (_)	_ _	_ _
v84c - v99c	Post	/ /	:	_ _ _	_ _ _	_ _ _	_ _ _	_ _ ._	_ _	_ _ _	_ _	_	_	_	_ _ (_)	_ _	_ _
v84d - v99d	Post	/ /	:	_ _ _	_ _ _	_ _ _	_ _ _	_ _ ._	_ _	_ _ _	_ _	_	_	_	_ _ (_)	_ _	_ _

V100

Capillary Blood Glucose *(TACPC ACR – CTPR. If both text and actual value are written, please choose “6. Specific value is provided” and transcribe the actual number below)*

1. “Normal”
2. “Low”
3. “High”
4. “CK Ketones”
5. No mention of capillary blood glucose
6. Specific value is provided (transcribe value below)

v101

↓ **If *Specific value,***

Capillary Blood Glucose – Actual Value

_____ _____ . _____ mmol/L

	**Co morbidities** *(TACPC ACR – Clinical Information – Relevant Past Hx or narrative sections. Box checked =1, narrative stating co morbidity present =1, box not checked =2, narrative co morbidity not present =3)*	
v102	**Previously Healthy** *(Previously Healthy box)*	**1** = Yes / **2** = Not documented on ACR
v103	**Cardiac** *(Cardiac box)*	**1** = Yes / **2** = Not documented on ACR / **3** = No
v104	**Respiratory** *(Respiratory box)*	**1** = Yes / **2** = Not documented on ACR / **3** = No
v105	**Stroke/TIA** *(Stroke/TIA box)*	**1** = Yes / **2** = Not documented on ACR / **3** = No
v106	**Hypertension** *(Hypertension box)*	**1** = Yes / **2** = Not documented on ACR / **3** = No
v107	**Seizure** *(Seizure box)*	**1** = Yes / **2** = Not documented on ACR / **3** = No
v108	**Diabetes** *(Diabetes box)*	**1** = Yes / **2** = Not documented on ACR / **3** = No
v109	**Psychiatric** *(Psychiatric box)*	**1** = Yes / **2** = Not documented on ACR / **3** = No
v110	**Other** *(Other box)*	**1** = Yes / **2** = Not documented on ACR / **3** = No
v111	**Cancer** *(Cancer box)*	**1** = Yes / **2** = Not documented on ACR / **3** = No
v112	**Other Co morbidity(s)** *(TACPC ACR – Clinical Information – Relevant Past History narrative)*	**1** = Yes / **2** = Not documented on ACR / **3** = No<br>↓ **If *Yes*,** please transcribe:

INTERVENTIONS - GENERAL

v113	**Oxygen Therapy Prior to Arrival** *(any documented oxygen therapy hook-up (successful and unsuccessful) from TACPC ACR Clinical Information – Treatment prior to arrival and ACR - CTPR (codes 130 – 142). If date spans midnight, confirm with CAD). See codes v117-119*	**1** = Yes / **2** = Not documented on ACR / **3** = No<br>↓ **If *Yes*,**
v116		**Prior Oxygen Therapy Code** *(the 1st oxygen therapy given from hook-up. If first and last then stop here)*<br>__ __ __ (unknown code = 999)<br>130 – 142
v116a	**Initial TACPC Oxygen Therapy Same as Prior Oxygen Therapy?** *(ACR - CTPR (codes 130 – 142). If no change stated, circle "1", previous oxygen therapy continues). See codes v117-119.*	**1** = Yes / **2** = No **3** = Not Applicable<br>↓ **If *No, or not applicable***
v116b - v116d		**Initial TACPC Oxygen Therapy Date, Time, Code**<br>__ __ __ __ / __ __ __ / __ __ __ __ : __ __ __ __ __<br>y y y y m m m d d h h m m 130 – 142
v117 - v119	**Oxygen Therapy Most Invasive Date, Time, & Code** *(if > 1 oxygen therapy and same kind then copy. If >1 and different, transcribe most invasive (Lowest Oxygen Nasal Cannula (132) < Oxygen Simple Face Mask (131) < Oxygen High Concentration Mask (130) < Oxygen BVM (141) < Oxygen (Mechanical) (142) Highest. If Oxygen Other (133), then decided via consensus. Time from hook-up If no change in oxygen therapy, leave blank.)*	__ __ __ __ / __ __ __ / __ __ __ __ : __ __ __ __ __<br>y y y y m m m d d h h m m 130 – 142

v120 **Immobilization** *(TACPC ACR Clinical Information – Treatment prior to arrival and ACR - CTPR (codes 105, 111, 112, 113, and 115))*

1 = Yes / **2** = Not documented on ACR / **3** = No

↓ **if *Yes*,**

v121 **Immobilization Head (105)**

1 = Yes / **2** = Not documented on ACR / **3** = No

v122 **Cervical Collar (111)**

1 = Yes / **2** = Not documented on ACR / **3** = No

v123 **Spinal Board (112)**

1 = Yes / **2** = Not documented on ACR / **3** = No

v124 **K.E.D (113)**

1 = Yes / **2** = Not documented on ACR / **3** = No

v125 **Scoop Stretcher (115)**

1 = Yes / **2** = Not documented on ACR / **3** = No

v126 **Other**

1 = Yes / **2** = Not documented on ACR / **3** = No

↓ **if *Yes*,** please specify:

v127 **Airway Management** *(airway management or airway breathing procedure from TACPC ACR Clinical Information – Treatment prior to arrival and ACR - CTPR (codes170, 171, 322, 324, and 326))*

1 = Yes / **2** = Not documented on ACR / **3** = No

↓ **if *Yes*,**

v128 **Airway Management Prior to Study Fluid**

(if 1 then transcribe type. If > 1 procedure ***prior to study fluid****, transcribe the most invasive. Lowest NPA (170) ORO/NPA (170) < OPA (170) < LMA (171) <Naso Tracheal Tube (324) < Oral Tracheal Tube (326) < Surgical (322) Highest)*

1. ORO / NASOPHARYNGEAL AIRWAY (NPA) (170)
2. LMA (171)
3. NASO-TRACHEAL INTUBATION (324)
4. ORO-TRACHEAL INTUBATION (326)
5. NEEDLE / SURGICAL CRICOTHYROIDOTOMY (322)
6. Other, please specify:
7. Not documented

v129 **Airway Management During or Post Study Fluid** *(if 1 then transcribe type. If > 1 procedure, transcribe the most invasive. Lowest NPA (170) ORO/NPA (170) < OPA (170) < LMA (171) <Naso Tracheal Tube (324) < Oral Tracheal Tube (326) < Surgical (322) Highest)*

1. No Change
2. ORO / NASOPHARYNGEAL AIRWAY (NPA) (170)
3. LMA (171)
4. NASO-TRACHEAL INTUBATION (324)
5. ORO-TRACHEAL INTUBATION (326)
6. NEEDLE / SURGICAL CRICOTHYROIDOTOMY (322)
7. Other, please specify:

v130 **Ventilation** *(TACPC ACR Clinical Information – Treatment prior to arrival and ACR - CTPR (codes 141, 142). If patient intubated circle "2" unless otherwise stated).*

1. No ventilation since patient breathing spontaneously
2. Yes, manual assist bag valve mask (BMV)
3. Yes, automatic assist (e.g., Genesis)
4. Not documented on ACR
5. Other, please specify:

v131

Chest Decompression Needle / Thoracostomy *(TACPC ACR Clinical Information – Treatment prior to arrival and ACR - CTPR (codes 320, 321))*

1. Yes (320)
2. Unsuccessful (321)
3. Both 320 and 321
4. Not documented on ACR
5. Other, please specify:

v132

Haemorrhage Control *(TACPC ACR Clinical Information – Treatment prior to arrival and ACR - CTPR (code 101))*

1 = Yes / 2 = Not documented on ACR / 3 = No

INTRAVENOUS (IV) FLUIDS RECEIVED

v133

IV Attempts *(total number of documented attempts (350) by all crews prior to IV access success according to TACPC ACR – Clinical Information – Treatment prior to arrival or CTPR or General Administration – Remarks / Orders. Three attempts is the maximum number of tries permitted)*

1. No documented unsuccessful attempt
2. One unsuccessful attempt (350) prior to access (2 attempts in all)
3. Two unsuccessful attempts (350) prior to access
4. Three unsuccessful attempts (350) prior to access
5. Other, please specify:

v134

IV Access - First to Achieve *(TACPC ACR –Clinical Information – Treatment prior to arrival or CTPR or General Administration – Remarks / Orders)*

1. Level II alone
2. Level I or II with preceptor
3. Prior ACP crew (that did not administer study fluid)
4. TOPHR HIT ACP Crew (TACPC)
5. Not Documented
6. Other EMS crew, please specify:
7. Other, please specify:

v135, v136

IV Access Date *(first date/time of successful IV access for any type of IV fluid (e.g., saline or hypertonic saline) by prior crew or TACPC according to TACPC ACR –Clinical Information – Treatment prior to arrival or CTPR or General Administration – Remarks / Orders. If date spans midnight, confirm with CAD).*

___ ___ ___ ___ / ___ ___ ___ / ___ ___
y y y y m m m d d

IV Access Time *(first documented time of successful IV access of any type of IV fluid (e.g., saline or hypertonic saline) by prior crew or TACPC according to TACPC ACR – Clinical Information – Treatment prior to arrival or CTPR or General Administration – Remarks / Orders)*

___ ___ : ___ ___ (00:00 (midnight) – 23:59)
h h m m

v137

IV Normal Saline Prior to Study Fluid *(the total amount of normal saline given to the patient by prior crew and/or TACPC prior to TOPHR HIT IV study fluid according to TACPC ACR – Clinical Information – Treatment prior to arrival or CTPR or General Administration – Remarks / Orders. Other than the study fluid that might be either normal saline or hypertonic saline, normal saline is the only type of IV fluid used by Toronto EMS. (Ringer's Lactate, D5W, 2/3-1/3, and Pentaspan are not used by TEMS). If other IV solution (349) is recorded, then what to do regarding the case must be decided via consensus and documented with a "note-to-file")*

1 = Yes / **2** = TKVO / **3** = Not Documented / **4** = None
↓ **if *Yes*,** fill in volume

Total Volume

____ ____ ____ ____ mL (≤ 9999 mL)

v140

IV Study Fluid Started *(IV study fluid was successfully started by the TOPHR HIT ACP crew – TACPC ACR CTPR. Note that "IV Study Fluid" is either hypertonic saline or normal saline.* ***If Study Fluid was not started, this is a MISSED CASE and should be assigned a Subject ID Code >=200)***

1 = Yes / **2** = Not documented on ACR / **3** = No
↓ **if *Yes*,** ***(If 2 or 3, skip to Prehospital Medications)***

v138

IV Study Fluid Randomization No. *(TOPHR HIT IV study fluid label from TACPC ACR (yellow copy) and/or from TPDC. If >1 label, decide on most appropriate randomization number via consensus and create a note-to-file)*

____ ____ ____ ____ (0001 – 9999)

v139

IV Study Fluid Type *(once the next study envelope in sequence is opened, the type of study fluid is no longer masked or 'blinded'. IV study fluid that was used for the patient according to the TACPC Crew Member 1 (Attendant) and available from the TPDC – IV Study Fluid Type))*

1 = HSD / **2** = Normal Saline (345)

v146

IV Study Fluid (HSD or Normal Saline) Total Volume *(total volume IV study fluid=HSD or NS received by patient on TACPC ACR –CTPR and from TPDC. A patient might receive up to 250 mL)*

___ ___ ___ mL (≤ 250 mL)

v141, v142

IV Study Fluid Start Date *(from date/time clamp opened according to TACPC ACR - CTPR. If date spans midnight, confirm. If more than one start and stop, take the first start and last stop. If study fluid was stopped and started more than once, take first start and last stop for start and stop date/time, respectively)*

___ ___ ___ ___ / ___ ___ ___ / ___ ___
y y y y m m m d d

IV Study Fluid Start Time

___ ___ : ___ ___ (00:00 (midnight) – 23:59)
h h m m

v143

IV Study Fluid Interrupted *(the study fluid was started by the TACPC and interrupted, however, the flow might have been reestablished and the patient might have received a complete infusion according to TACPC ACR Clinical Information –CTPR or General Administration – Remarks/Orders and from TPDC)*

1 = Yes / **2** = Not documented on ACR
↓ **if *Yes*,** please specify:

v144

IV Study Fluid Complete Infusion (250 mL)
(250mL were completely received by the patient according to TACPC ACR Clinical Information – CTPR or General Administration – Remarks/Orders and from TPDC)

1 =Yes (250mL) / **2** =No / **3** = Not documented on ACR

↓ **If *No*,**

v145

Reason for Incomplete Infusion of Study Fluid *(the study fluid (either hypertonic saline or normal saline) was started by the TACPC, and 250mL were not completely received by the patient. Record reason according to TACPC ACR Clinical Information –CTPR and from TPDC)*

1. IV stopped due to poor connection
2. IV pulled out by patient
3. IV interstitial
4. IV stopped by hospital staff
5. IV stopped due to **Adverse Drug Reaction (ADR)[1] to HSD**, please specify:
6. Other, please specify:

v145a, v145b

IV Study Fluid Stop Date *(any corresponding documented IV study fluid from the date/time that the clamp was closed on TACPC ACR-CTPR and from TPDC. If date spans midnight confirm with CAD. If more than one start and stop, take first start and last stop)*

___ ___ ___ ___/___ ___ ___/___ ___
y y y y m m m d d

IV Study Fluid Stop Time *(any corresponding documented IV study fluid from the time that the clamp was closed on TAPCR ACR Clinical Information –CTPR and from TPDC.)*

___ ___:___ ___ (00:00 (midnight)-23:59)
h h m m

v148

IV Normal Saline Total Volume Post Study Fluid *(total volume post IV study fluid from study fluid stop to ED from TACPC ACR –CTPR and & TPDC)*

1 = Yes Volume Given /**2** = TKVO / **3** = Not Documented
↓ **if *Yes*,** fill in volume

___ ___ ___ ___ mL (≤ 9999 mL)

[1] Adverse Drug Reaction (ADR) defined as all noxious and unintended responses to Hypertonic Saline in Dextrose (HSD) at any dose (Health Canada)

PARAMEDIC IMPRESSION OF STUDY FLUID

v149	**Ease of Use** *(ease of IV study fluid use according to crew member 1 (Attendant) paramedic from TPDC – The study fluid is easy to administer)*	**1** (agree) **2** **3** **4** **5** (disagree)
v150	**Medical Directive Compliance** *(ease of TOPHR HIT medical directive according to TACPC crew member 1 (Attendant) paramedic from TPDC – The medical directive is easy to follow)*	**1** (agree) **2** **3** **4** **5** (disagree)
v151	**Paramedic Hesitation to Use of Hypertonic Saline in 6% Dextran (HSD)** *(TPDC–Do you have any hesitation to using HSD?)*	**1** = Yes / **2** = No / **3** = Not documented on ACR<br>↓ **if *Yes*,** please specify:

PREHOSPITAL MEDICATIONS

v152	**Morphine (604)** *(TACPC ACR – CTPR)*	**1** = Yes / **2** = Not documented on ACR / **3** = No
v153	**Midazolam (603)** *(TACPC ACR – CTPR)*	**1** = Yes / **2** = Not documented on ACR / **3** = No
v154	**Lidocaine (591)** *(TACPC ACR – CTPR)*	**1** = Yes / **2** = Not documented on ACR / **3** = No
v155	**Lidocaine Topical (593)** *(TACPC ACR – CTPR)*	**1** = Yes / **2** = Not documented on ACR / **3** = No
v156	**Diazepam or Valium (531)** *(TACPC ACR – CTPR)*	**1** = Yes / **2** = Not documented on ACR / **3** = No
v157	**D50W (530)** *(TACPC ACR – CTPR)*	**1** = Yes / **2** = Not documented on ACR / **3** = No
v158	**Glucagon (560)** *(TACPC ACR – CTPR)*	**1** = Yes / **2** = Not documented on ACR / **3** = No
v159	**Naloxone (610)** *(TACPC ACR – CTPR)*	**1** = Yes / **2** = Not documented on ACR / **3** = No
v160	**Mannitol (601)** *(TACPC ACR – CTPR)*	**1** = Yes / **2** = Not documented on ACR / **3** = No
v161	**Succinylcholine or Anectine (655)** *(TACPC ACR – CTPR)*	**1** = Yes / **2** = Not documented on ACR / **3** = No
v162	**Vecuronium or Norcuron (680)** *(TACPC ACR – CTPR)*	**1** = Yes / **2** = Not documented on ACR / **3** = No
v163	**Other** *(TACPC ACR – CTPR)*	**1** = Yes / **2** = Not documented on ACR / **3** = No<br>↓ **If *Yes*,** please specify:

PREHOSPITAL OUTCOME

v164 **Pronounced on Scene** *(pronounced by Base Hospital Physician while still at scene (code=366) according to TACPC ACR –CTPR or General Administration – Remarks/Orders)*

1 = Yes, patient pronounced at scene | **2** = No, patient not pronounced at scene

↓ **If *Yes***

v165 v167 **Date Pronounced**

___ ___ ___ ___ / ___ ___ ___ / ___ ___
y y y y m m m d d

v166 **Time Pronounced**

____ ____ : ____ ____ (00:00 – 23:59)
h h m m **<STOP>**

↓ **If *No***

CTAS Score (Depart Scene)
(TPDC – CTAS Score (depart scene))

1. Resuscitation
2. Emergency
3. Urgent
4. Less Urgent
5. Non-urgent
6. Missing (from TPDC)

v168 **Pronounced Prior to Arrival at Hospital** *(pronounced by Base Hospital Physician prior to arrival at SWCHSC or SMH (code=366) according to TACPC ACR – CTPR or General Administration – Remarks/Orders. VSA en route is not the same as pronounced en route)*

1 = Yes (pronounced prior to arrival) | **2** =No (*not* pronounced prior to arrival)

↓ **If *Yes***

v169 v171 **Date Pronounced**

___ ___ ___ ___ / ___ ___ ___ / ___ ___
y y y y m m m d d

v170 **Time Pronounced**

____ ____ : ____ ____ (00:00 – 23:59)
h h m m **<STOP>**

↓ **If *No***

CTAS Score *(TACPC TPDC – CTAS Score (arrive SWCHSC or SMH hospital))*

1. Resuscitation
2. Emergency
3. Urgent
4. Less Urgent
5. Non-urgent
6. Missing (from TPDC)

v172 **Patient Status Change Score** *(TPDC – Patient Status Change Score at arrival to SWCHSC or SMH)*

1. Improved
2. Unchanged
3. Worsened
4. Became VSA en route
5. Missing (from TPDC)

v173 **Receiving Trauma Facility** *(This field must still be completed for survivors as well as DOAs, as long as they were not pronounced en route. Name of the hospital that the unpronounced patient was brought to by the TACPC. TACPC ACR - General Administration – Hospital No. or Receiving facility/destination)*

1. Sunnybrook & Women's
2. St. Michael's Hospital

v32a **Pronounced in Emerg?** *(TACPC ACR - General Administration – Pt. Outcome in Emerg.— 'Pronounced' checkbox)*
1 = Yes / **2** = Not Documented / **3** = No

v176 **Diversion or Transfer** *(TACPC – Diversion or Transfer Prior to Receiving Trauma Facility)*
1 = Yes / **2** = Not documented / **3** = No
↓ **If *Yes***, please specify:

ABSTRACTION DETAILS

v176b **Enrollment Status**
*(Possible values are **Enrolled = "E", Missed Case = "M", Excluded = "X"**. This field will be automatically populated based on Inclusion Criteria, Exclusion Criteria and Study Fluid Administration fields. Check to ensure data entry system populates with the correct value; override the system generated value if necessary and contact the study administrator.)*

______ (E, M, or X)

v177 **Paramedic Research Coordinator Initials**

______ ______
first last

v178 **Date Paramedic Research Coordinator Abstraction**

___ ___ ___ ___ / ___ ___ ___ / ___ ___
y y y y m m m d d

INVESTIGATOR STATEMENT

I certify that: I have carefully examined and verified all entries in this case form. All information entered onto these forms by myself and/or my associates is correct.

Principle Investigator Signature (LJM): ____

Date of Principle Investigator Verification (LJM):

___ ___ ___ ___ / ___ ___ ___ / ___ ___
y y y y m m m d d
