# Supplementary material for: The Toronto prehospital hypertonic resuscitation-head injury and multi organ dysfunction trial (TOPHR HIT) - Methods and data collection tools
Source: Trials. 2009 Nov 20;10:105. doi: 10.1186/1745-6215-10-105 (PMC2788534; doi:10.1186/1745-6215-10-105)
Supplement: Additional file 13 — In-hospital case report form. [file 1745-6215-10-105-S13.PDF]

THE TORONTO PREHOSPITAL HYPERTONIC RESUSCITATION - HEAD INJURY AND MULTI ORGAN DYSFUNCTION FEASIBILITY TRIAL (TOPHR HIT)
INHOSPITAL SECURE PATIENT INFORMATION FORM

Tyrone Perreira B.Sc., ACP, Paramedic Research Coordinator, Prehospital and Transport Medicine Research Program, SWCHSC Tel: 416-480-6100x7072 Email: tyrone.perreira@sw.ca

Note: The current page is highly confidential, will be used for administrative purposes only, and must be kept secure

DEMOGRAPHICS

For admin. purposes only

Name

(Surname) (First name) (Middle name)

Address

(Street Number) (Street) (Unit/Apt)

(City) (Province) (Postal Code)

Phone 1 *(at arrival to ED)*

Home: (___ ___ ___) ___ ___ ___ - ___ ___ ___ ___ Other 1: (___ ___ ___) ___ ___ ___ - ___ ___ ___ ___

Work: (___ ___ ___) ___ ___ ___ - ___ ___ ___ ___ Other 2: (___ ___ ___) ___ ___ ___ - ___ ___ ___ ___

Phone 2 *(at 4 and 12 months if different)*

Home: (___ ___ ___) ___ ___ ___ - ___ ___ ___ ___ Other 1: (___ ___ ___) ___ ___ ___ - ___ ___ ___ ___

Work: (___ ___ ___) ___ ___ ___ - ___ ___ ___ ___ Other 2: (___ ___ ___) ___ ___ ___ - ___ ___ ___ ___

Next of Kin 1

(Surname) (First name)

Home: (___ ___ ___) ___ ___ ___ - ___ ___ ___ ___ Other 1: (___ ___ ___) ___ ___ ___ - ___ ___ ___ ___

Work: (___ ___ ___) ___ ___ ___ - ___ ___ ___ ___ Other 2: (___ ___ ___) ___ ___ ___ - ___ ___ ___ ___

Next of Kin 2

(Surname) (First name)

Home: (___ ___ ___) ___ ___ ___ - ___ ___ ___ ___ Other 1: (___ ___ ___) ___ ___ ___ - ___ ___ ___ ___

Work: (___ ___ ___) ___ ___ ___ - ___ ___ ___ ___ Other 2: (___ ___ ___) ___ ___ ___ - ___ ___ ___ ___

Date of birth ___ ___ ___ ___ / ___ ___ ___ / ___ ___
y y y y m m m d d

Gender (**M**)ale / (**F**)emale / (**U**)known / (**N**)ot available

HIN ___ ___ ___ ___ ___ ___ ___ ___ ___ ___

HIN Version Code ____ ____

Hospital Name **1**. SMH / **2**. SWCHSC / **3**. Other, please specify:

Hospital Chart No. *(SMH "J" or SWCHSC "HFN" chart. Include the "J" or "HFN")* ___ ___ ___ ___ ___ ___ ___ ___ ___ ___

CONSENT

Consent Obtained *(from patient or patient's next of kin, if the patient was not conscious, by inhospital Research Assistant)* **1** = Yes / **2** = No

Consent Obtained From *(if "Yes", record who provided the consent)* **1**. Patient / **2**. Next of Kin / **3**. Partner or Spouse / **4**. Other:

Consent Obtained Date *(if "Yes", date and time obtained consent)*

____ ____ ____ ____ / ____ ____ ____ / ____ ____

Consent Obtained Time

___ ___ : ___ ___ (00:00:00 (midnight) – 23:59:59)
h h m m

CALL INFORMATION & RANDOMIZATION NUMBER

Call Number *(EMS call no. from ACR "Administration – Call Number/Patient Number". The ACR must be from the ACP crew that administered the study fluid and transported the patient (i.e., "**treating ACP crew (TACPC)**")*

___ ___ ___ ___ ___ ___ ___ ___ ___ ___ ___ ___

Call Received Date *(TACP ACR Administration - Call Date (YMD))*

____ ____ ____ ____ / ____ ____ ____ / ____ ____
y y y y m m m d d

Call Received Time *(TACPC ACR – Call Events - Call Received)*

___ ___ : ___ ___ : ___ ___ (00:00:00 (midnight) – 23:59:59)
h h m m s s

Crew Member 1 (Attendant) No. *(TACPC OASIS # from ACR General Administration – Crew Member 1 (Attendant) No.)*

____ ____ ____ ____ ____ ____

IV Study Fluid Randomization No. *(sticker from TACPC ACR (white copy). **If no sticker or >1 sticker, notify Tyrone asap (W)416-480-6100x7072)***

____ ____ ____ ____ (0001 – 9999)

INCLUSION CRITERIA

Age ≥16 years *((TACPC ACR Administration – Date of Birth (YMD) or estimated age from ACR – Physical Exam – Age (must be ≥ 16 years) and **TOPHR HIT Paramedic Data Checklist** (**TPDC**) – Inclusion Criteria – Age 0 16. All discrepancies must be resolved prior to Study ID Code assignment)*

1 = Yes / **2** = No

Blunt Traumatic Injury *((TACPC ACR –Clinical Information – Traumatic Injury Site/Type - Type column=34 or 35 or Incident History or General Information-Remarks) and TPDC–Inclusion Criteria –Blunt Trauma. Note "crush injury" is considered to be a form of blunt trauma here)*

1 = Yes / **2** = No

GCS *(total GCS score immediately prior to study fluid from TACPC ACR - Clinical Treatment/Procedures & Results (**CTPR**) and TPDC – Inclusion Criteria - GCS≤8. To be included total score must range from 03 - ≤8)*

1 = Yes / **2** = No

ACP Treated, Transported, and ACR *(one ACP crew administered the study IV fluid and the same ACP crew transported the patient (if the patient was not pronounced at scene) and completed the ACR, which was used for this CRF as the original source document (ACR yellow copy). Aka TACPC for "treating ACP crew". And the TPDC - TACPC treated, transported, and ACR boxes are checked. Level I or II precepting as Level III (with a preceptor) are included. Helicopter transport cases are decided via consensus)*

1 = Yes / **2** = No

IV Access *((IV access obtained at any point during the call TACPC ACR – CTPR - IV Procedures Codes or text (e.g., "IV running well" or "IV TKVO")) and TPDC – IV Access. Do not use top right Call Type "□ IV ONLY" since call limited to only IV given. IV codes include 340 and 345 (Normal Saline)))*

1 = Yes / **2** = No

EXCLUSION CRITERIA

Known or Suspected Pregnancy *(Hospital chart. Not using TACPC ACR unless necessary since no pertinent negatives are systematically recorded on ACR)* — **1** = Yes / **2** = No

Primary Injury Penetrating *(Hospital chart)* — **1** = Yes / **2** = No

VSA Prior to Randomization *(Hospital chart)* — **1** = Yes / **2** = No

Arrival at scene to IV access >4 hours *(Hospital chart)* — **1** = Yes / **2** = No

Amputation above wrist or ankle *(Hospital chart)* — **1** = Yes / **2** = No

Any burn *(Hospital chart)* — **1** = Yes / **2** = No

Suspected hypothermia *(Hospital chart)* — **1** = Yes / **2** = No

Asphyxia *(Hospital chart)* — 1 = Yes / 2 = No

Minor Fall *(no minor fall, i.e., no fall from standing on floor, no fall from height ≤ 1 m, or no fall from ≤ 5 stairs (TACPC ACR or Hospital chart))* — 1 = Yes / 2 = No

THE TORONTO PREHOSPITAL HYPERTONIC RESUSCITATION - HEAD INJURY AND MULTI ORGAN DYSFUNCTION FEASIBILITY TRIAL (TOPHR HIT) INHOSPITAL CASE REPORT FORM (FORM 2/4)

Tyrone Perreira B.Sc., ACP, Paramedic Research Coordinator, Prehospital and Transport Medicine Research Program, SWCHSC Tel: 416-480-6100x7072 Email: tyrone.perreira@sw.ca

DEMOGRAPHICS

row 1<br>v1, 1-11	**Date of birth** *(from patient or patient representative. If not possible, then from TACPC ACR Administration – Date of Birth (YMD). Must be ≥ 16 years)*	___ ___ ___ ___ / ___ ___ ___ / ___ ___<br>y y y y m m m d d
v2, 12	**Gender** *(from patient or patient representative. If not possible, TACPC ACR – Physical Exam - Gender)*	(M)ale / (F)emale / (U)known / (N)ot available
v3, 13-15	**Estimated Weight** *(to nearest kilogram with all heavy outdoor garments and shoes removed from patient or patient representative. If not possible, TACPC ACR – Physical Exam – Weight (kg). If recorded in pounds, Paramedic Research Coordinator to enter the equivalent in kilograms (kgs = pounds/2.2). Pad with zeros if necessary.)*	___ ___ ___ kg (approx. 040 kg – 250 kg)
v4, 16-18	**Estimated Height** *(to nearest centimeter or feet and inches without shoes from patient or patient representative)*	___ ___ ___ cms

CONSENT

v5, 19	**Consent Obtained** *(Amanda (SMH) or Sue (SWCHSC) will consent the patient, or the patient's next of kin if the patient is not conscious, using the SMH and SWCHSC consent forms, respectively)*	1 = Yes / 2 = No

CALL INFORMATION & RANDOMIZATION NUMBER

v6, 20-31	**Call Number** *(EMS call number as entered into the "Administration" section–"Call Number/Patient Number" field from the ACR that was completed by the ACP crew that administered the study fluid and transported the patient (i.e., "**treating ACP crew (TACPC)**". The ACR call information must be identical to the TOPHR HIT Data Checklist call information (e.g., Call #))*	___ ___ ___ ___ ___ ___ ___ ___ ___ ___ ___ ___
v7,v8<br>32-50	**Call Received Date** *(treating ACP crew ACR Administration - Call Date (YMD))*<br>___ ___ ___ ___ / ___ ___ ___ / ___ ___<br>y y y y m m m d d	**Call Received Time** *(TACPC ACR – Call Received)*<br>___ ___ : ___ ___ : ___ ___ (00:00:00 (midnight) – 23:59:59)<br>h h m m s s
v9<br>51-56	**Crew Member 1 (Attendant) No.** *(TACPC OASIS # from ACR General Administration – Crew Member 1 (Attendant) No.)*	___ ___ ___ ___ ___ ___
v10<br>57-60	**IV Study Fluid Randomization No.** *(IV fluid study label from TACPC ACR (white copy). **If no sticker or >1 sticker, pls notify Tyrone immediately (W)416-480-6100x7072**)*	___ ___ ___ ___ (0001 – 9999)

PATIENT HISTORY

Co morbidities *(co morbidity(s) present **before** infusion of test solution. From patient or patient representative, hospital chart, or TACPC ACR – Clinical Information – Relevant Past Hx - check boxes are marked. "No" means there is specific mention of no co morbidity, e.g., "no history of hypertension")*

v11, 61	**Previously Healthy** *(i.e., noted as no known co morbidity)*	1 = Yes / 2 = Not documented / 3 = No
v12, 62	**Cardiac**	1 = Yes / 2 = Not documented / 3 = No
v13, 63	**Grade 4 CHF**	1 = Yes / 2 = Not documented / 3 = No
v14, 64	**Stroke/TIA**	1 = Yes / 2 = Not documented / 3 = No
v15, 65	**Hypertension**	1 = Yes / 2 = Not documented / 3 = No
v16, 66	**Pulmonary**	1 = Yes / 2 = Not documented / 3 = No

v17, 67	**Respiratory**	**1** = Yes / **2** = Not documented / **3** = No
v18, 68	**Metabolic**	**1** = Yes / **2** = Not documented / **3** = No
v19, 69	**Liver Disease**	**1** = Yes / **2** = Not documented / **3** = No
v20, 70	**Dialysis Dependent Renal Disease**	**1** = Yes / **2** = Not documented / **3** = No
v21, 71	**Seizure**	**1** = Yes / **2** = Not documented / **3** = No
v22, 72	**Neurological Disease**	**1** = Yes / **2** = Not documented / **3** = No
v23, 73	**Coagulopathy**	**1** = Yes / **2** = Not documented / **3** = No
v24, 74	**Leukemia**	**1** = Yes / **2** = Not documented / **3** = No
v25, 75	**Cancer**	**1** = Yes / **2** = Not documented / **3** = No
v26, 76	**Radiation or Chemotherapy**	**1** = Yes / **2** = Not documented / **3** = No
v27, 77	**Anemia**	**1** = Yes / **2** = Not documented / **3** = No
v28, 78	**AIDS**	**1** = Yes / **2** = Not documented / **3** = No
v29, 79	**Diabetes**	**1** = Yes / **2** = Not documented / **3** = No
v30, 80	**Psychiatric**	**1** = Yes / **2** = Not documented / **3** = No
v31, 81	**Drug Dependency**	**1** = Yes / **2** = Not documented / **3** = No
v32, 82	**Previous Suicide Attempt**	**1** = Yes / **2** = Not documented / **3** = No
v33, 83	**Other Co morbidity(s)**	**1** = Yes / **2** = Not documented / **3** = No<br>↓ If *Yes*, please transcribe:

CIRCUMSTANCES OF INJURY

row 2

v34, 1 **Type of Injury** *(from patient or patient representative. If not possible, TACPC ACR –Clinical Information – Traumatic Injury Site/Type - Type column=34 or 35 or Incident History or General Information - Remarks)*

1. Blunt (34) / **2**. Crush (35) / **3**. Both / **4**. Other, specify:

v35, 2 **Mechanism of Injury** *(if >1 then the mechanism immediately prior to injury. From patient or patient representative. If not possible, TACPC ACR –Clinical Information – Traumatic Injury Site/Type – Mechanism column and TPDC – Basics of Mechanism)*

1.Transport / **2**.Fall / **3**.Interpersonal Violence / **4**.Other, specify:
58,59,60,61 / 53,54 / 50,55,63 / 51,52,56,57,62,64,65

↓ If *Transport,*

v36, 3 **Type of Transport** (If *Transport*, then please specify type of transport)

1. MVC (car or truck (58))
2. MOTORCYCLE/RECREATIONAL VEHICLE (59)
3. PEDAL BICYCLE (60)
4. PEDESTRIAN STRUCK (61)
5. Missing
6. Other, please specify:

v37, 4 **Location of Injury** *(from patient or patient representative. If not possible, TACPC ACR. Urban is defined as the City of Toronto, which is made up of the following 6 Municipalities: Toronto, East York, York, Etobicoke, North York, and Scarborough. Rural is defined as the municipalities outside of the City of Toronto but within the Greater Toronto Area (GTA) Regions of Halton, Peel, York, and Durham. For a full list of the GTA Municipalities see http://www.greater.toronto.on.ca/index.html (last accessed September 15, 2004))*

1. Urban (City of Toronto)
2. Rural (Greater Toronto Area (GTA) but not City)
3. Remote (outside of GTA)
4. Unknown
5. Other, please specify:

v38, 5 **Deliberate Self-Harm** *(related to current circumstance of injury. From patient or patient representative. TACPC ACR Clinical Information – Incident History)*

1=Yes / **2**=Suspected / **3**=Not documented / **4**=No

EMERGENCY DEPARTMENT (ED)

v39, 6

Receiving Trauma Hospital ED *(the first and only receiving hospital must be either SMH or SWCHSC. If the study criteria are met and the patient was not "pronounced in the field" and brought to a non study trauma centre, then the patient is not considered eligible for the TOPHR HIT. Transcribe name from TACPC ACR - General Administration – Hospital No. or Receiving facility/destination)*

1. St. Michael's Hospital
2. SWCHSC
3. Other, please specify:

v40, v41
7-22

Arrival to ED Date *(according to 'Admission Form')*

___ ___ ___ ___ / ___ ___ ___ / ___ ___
y y y y m m m d d

Arrival to ED Time *(according to 'Admission Form')*

___ ___ : ___ ___ (00:00:00 (midnight) – 23:59:59)
h h m m

v42, 23

Post ED status *(if the patient died in ED then use this form to document what happened in the ED and be sure to complete the Hospital Outcome section. If the patient was transferred at any time, again use this form to document the current stay and complete the Hospital Outcome section).*

1. Admitted to floor or unit of same hospital
2. Died in ED *(record results and Hospital Outcome)*
3. Transferred to another hospital:

Hospital's' name: ____ ____ ____ ____ ____ ____ ____
City: ____ ____ ____ ____ ____ ____

4. Other, please specify:

HOSPITAL ADMISSION INFORMATION

v43, 24

Receiving Trauma Hospital *(if the patient was not pronounced in the ED and was admitted to hospital)*

1. St. Michael's Hospital
2. SWCHSC
3. Other, please specify:

v44, v45
25-40

Receiving Trauma Hospital Admission Date *(according to the 'In-patient Admission Record')*

___ ___ ___ ___ / ___ ___ ___ / ___ ___
y y y y m m m d d

Receiving Trauma Hospital Admission Time *(according to the 'In-patient Admission Record')*

___ ___ : ___ ___ (00:00:00 (midnight) – 23:59:59)
h h m m

v46, 41

Admitted from *(where the patient was admitted from shortly after arrival according to the 'In-patient Admission Record')*

1. Emergency Department (ED)
2. Transferred from another hospital's ED, specify:
3. Transferred from another hospital as an in-patient, specify:
4. Other, please specify:

v47, 42

Admitted to *(service or unit where the patient was admitted to shortly after arrival according to the 'In-patient Admission Record' or 'Doctor's Admission Note')*

1. Trauma/Intensive Care Setting (e.g., TNICU or ICU)
2. Hospital Ward
3. Another acute care facility *(please complete Form A)*
4. Other *(please complete Form A)*

TRAUMA/INTENSIVE CARE SETTING (TNICU, NICU,CCU, ICU, MICU, CRCU, SICU)

v48, 43

Admitted *(admitted to an intensive care unit (e.g., TNICU, NICU, CCU, ICU, MICU, CRCU, or SICU), including initial admission, according to 'Emergency Nursing Notes', 'Doctor's Orders' ("transfer to" or "admit to" notes), 'Doctor's Notes', and/or 'Cardiac/Intensive Care Unit Nursing Notes'. Note that a transfer from one Trauma/Intensive Care Unit to another within the same hospital is* ***not*** *considered to be a discharge and admission, respectively. If a patient is admitted to an intensive care unit more than twice, please complete a* ***TOPHR HIT Trauma/Intensive Care Setting Form (Form B)*** *for each additional admission)*

1. No, not admitted to a NICU, CCU, ICU, MICU, CRCU, or SICU
2. Yes, Admitted Once
3. Yes, Admitted Twice (i.e., admitted-discharged-admitted)
4. Yes, Admitted Three times or more, *please complete Form B for each additional admission and specify the number of admissions here:* ___ ___
5. Not documented in chart

6. Other, please specify:

row3 v49 1-2
row4 v60 1-2

Admission number **0 1**

v50, 3-13
v61, 3-13

Patient Received Date *(date of "patient received" in Nursing Notes)*

___ ___ ___ ___ / ___ ___ ___ / ___ ___
y y y y m m m d d

v51, 14-18
v62, 14-18

Patient Received Time *(from 'Nursing Notes')*

___ ___ : ___ ___ (00:00:00 (midnight) – 23:59:59)
h h m m

v52, 19-20
v63, 19-20

APACHE II *(please use the **APACHE II Form (Form C)** to score APACHE II once at admission to intensive care setting and enter the total score below)*

____ ____

v53, 21-22
v64, 21-22

SOFA *(please use **the SOFA Form (Form D)** to score SOFA once at admission to intensive care setting and enter the total score below. **If applicable, please complete a Form D for every 2nd day while the patient is in the intensive care setting until death, discharge, or transfer (starting from 48 hours from arrival). Attached all forms to the CRF and submit together)***

____ ____

v54, 23-24
v65, 23-24

MODS *(please use the **MODS Form (Form E**) to score MODS once at admission to intensive care setting and enter the total score below. **If applicable, please complete a Form E for every 2nd day while the patient is in the intensive care setting until death, discharge, or transfer (starting from 48 hours from arrival). Attached all forms to the CRF and submit together)***

____ ____

v55, 25-26
v66, 25-26

Total number of days in intensive care unit *(total number of days in intensive care until death of discharge or transfer from intensive care)*

____ ____

v56, 27
v67, 27

Survived to 1st intensive care setting release *(survived to discharge or transfer from intensive care setting (e.g., TNICU, CCU, ICU, MICU, CRCU, or SICU) according to Doctor's Orders ("transfer to" or "admit to") and/or Nursing Notes ("receive from"))*

1. Yes, survived to release from trauma/intensive care unit

2. No, died in trauma/intensive care unit - *record results and Hospital Outcome*

3. No, still in at time of abstraction

4. Not documented in chart

5. Other, please specify:

v57, 28-38
v68, 28-38

Patient Release Date *(if survived to end of intensive care stay, discharge or transfer from intensive care setting to another floor, unit, or facility, not including death, according to Doctor's Orders ("transfer to" or "admit to") and/or Nursing Notes ("received from"))*

___ ___ ___ ___ / ___ ___ ___ / ___ ___
y y y y m m m d d

v58, 39-43
v69, 39-43

Patient Release Time *(if survived to end of intensive care stay, from 'Nursing Notes')*

___ ___ : ___ ___ (00:00:00 (midnight) – 23:59:59)
h h m m

Admission number **0 2**

Patient Received Date

___ ___ ___ ___ / ___ ___ ___ / ___ ___
y y y y m m m d d

Patient Received Time *(from 'Nursing Notes')*

___ ___ : ___ ___ (00:00:00 (midnight) – 23:59:59)
h h m m

APACHE II *(please use the **APACHE II Form (Form C)** to score APACHE II once at admission to intensive care setting and enter the total score below)*

____ ____

SOFA *(please use **the SOFA Form (Form D)** to score SOFA once at admission to intensive care setting and enter the total score below. **If applicable, please complete a Form D for every 2nd day while the patient is in the intensive care setting until death, discharge, or transfer (starting from 48 hours from arrival). Attached all forms to the CRF and submit together)***

____ ____

MODS *(please use the **MODS Form (Form E**) to score MODS once at admission to intensive care setting and enter the total score below. **If applicable, please complete a Form E for every 2nd day while the patient is in the intensive care setting until death, discharge, or transfer (starting from 48 hours from arrival). Attached all forms to the CRF and submit together)***

____ ____

Total number of days in intensive care unit *(total number of days in intensive care until death of discharge or transfer from intensive care)*

____ ____

Survived to 2nd intensive care setting release *(survived to discharge or transfer from intensive care setting (e.g., TNICU, CCU, ICU, MICU, CRCU, or SICU) according to Doctor's Orders ("transfer to" or "admit to") and/or Nursing Notes ("receive from"))*

1. Yes, survived to release from trauma/intensive care unit

2. No, died in trauma/intensive care unit - *record results and Hospital Outcome*

3. No, still in at time of abstraction

4. Not documented in chart

5. Other, please specify:

Patient Release Date *(if survived to end of intensive care stay, discharge or transfer from intensive care setting to another floor, unit, or facility, not including death, according to Doctor's Orders ("transfer to" or "admit to") and/or Nursing Notes ("received from"))*

___ ___ ___ ___ / ___ ___ ___ / ___ ___
y y y y m m m d d

Patient Release Time *(if survived to end of intensive care stay, from 'Nursing Notes')*

___ ___ : ___ ___ (00:00:00 (midnight) – 23:59:59)
h h m m

v59, 44<br>*v80, 44*	**Release To** *(if survived to end of intensive care stay, from 'Admitting Notes' or admitting patient tracking system)*<br>**1.** Hospital Ward<br>**2.** Another acute care hospital, *please fill in Form A*<br>**3.** Non acute extended or chronic care facility, *please fill in Form A*<br>**4.** Home<br>**5.** Left against medical advice (AMA)<br>**6.** Not documented in the chart<br>**7.** Other, please specify:	**Release To** *(if survived to end of intensive care stay, from 'Admitting Notes' or admitting patient tracking system)*<br>**1.** Hospital Ward<br>**2.** Another acute care hospital, *please fill in Form A*<br>**3.** Non acute extended or chronic care facility, *please fill in Form A*<br>**4.** Home<br>**5.** Left against medical advice (AMA)<br>**6.** Not documented in the chart<br>**7.** Other, please specify:

PATIENT ASSESSMENT

		**Arrival to ED** *(first set of vitals obtained on the patient upon arrival to ED. Transcribe date/time from previous section for your conveniences)*<br>_ _ _ _ / _ _ _ / _ _ __ __ : __ __ (24 hr)<br>y y y y m m m d d h h m m	**Post Arrival to ED ≥60 mins to ≤120 mins**<br>_ _ _ _ / _ _ _ / _ _ __ __ : __ __ (24 hr)<br>y y y y m m m d d h h m m
row 5<br>v81-v82,<br>1-2	**Glasgow Coma Scale (GCS)**[1]<br>**Eye Opening** *(according to 'Emergency Records' or 'TTL Notes')*	**1.** None<br>**2.** To Pain<br>**3.** To Voice<br>**4.** Spontaneous	**1.** None<br>**2.** To Pain<br>**3.** To Voice<br>**4.** Spontaneous
v83-v84,<br>3-4	**GCS Verbal Response** *(according to 'Emergency Records' or 'TTL Notes')*	**1.** None<br>**2.** Incomprehension. Sounds<br>**3.** Inappropriate Words<br>**4.** Confused<br>**5.** Oriented	**1.** None<br>**2.** Incomprehension. Sounds<br>**3.** Inappropriate Words<br>**4.** Confused<br>**5.** Oriented
v85-v86,<br>5-6	**GCS Motor Response** *(according to 'Emergency Records' or 'TTL Notes')*	**1.** None<br>**2.** Extension (pain)<br>**3.** Flexion (pain)<br>**4.** Withdraw (pain)<br>**5.** Localize (pain)<br>**6.** Obeys Commands	**1.** None<br>**2.** Extension (pain)<br>**3.** Flexion (pain)<br>**4.** Withdraw (pain)<br>**5.** Localize (pain)<br>**6.** Obeys Commands
v87-v88,<br>7-10	**Respiration Rate** *(according to 'Emergency Records' or 'TTL Notes')*	____ ____ per min	____ ____ per min
v89-v90,<br>11-16	**Heart Rate** *(according to 'Emergency Records' or 'TTL Notes')*	____ ____ ____ beats per min	____ ____ ____ beats per min
v91-v92,<br>17-22	**Systolic Blood Pressure** *(according to 'Emergency Records' or 'TTL Notes')*	____ ____ ____ mmHg	____ ____ ____ mmHg
v93-v94,<br>23-28	**Diastolic Blood Pressure** *(according to 'Emergency Records' or 'TTL Notes')*	____ ____ ____ mmHg	____ ____ ____ mmHg
v95-v96,<br>29-34	**Pulse Oximetry** *('ED Records' or 'TTL')*	____ ____ ____ %	____ ____ ____ %
v97-v98,<br>35-42	**Temperature** *('ED Records' or 'TTL')*	____ ____ . ____ ºC	____ ____ . ____ ºC
v99, 43	**Head CT Scan**	**1**=Yes / **2**=Suspected / **3**=Not doc. / **4**=No<br>↓If *Yes,*	
v100, 44-54		**Head CT Scan Date**<br>___ ___ ___ ___ / ___ ___ ___ /___ ___<br>y y y y m m m d d	
v101, 55-59		**Head CT Scan Time**	

[1] Lancet. 1974 Jul 13;2(7872):81-4

		___ ___ : ___ ___<br>h h m m	
v102, 60		**Head CT Scan Category**[2] *(transcribe from 'Neurosurgery Diagnostic Results')*<br>**1.** Diffuse Injury I *(no visible intracranial pathology seen on CT scan)*<br>**2.** Diffuse Injury II *(cisterns are present, with midline shift 0-5 mm, and/or there is no high or mixed density lesion >25cc)*<br>**3.** Diffuse Injury III *(cisterns compressed or absent with midline shift 0-5 mm, no high or mixed density lesion >25cc)*<br>**4.** Diffuse Injury IV *(midline shift >5 mm, no high or mixed density lesion >25cc)*<br>**5.** Mass Lesion *(any lesion surgically evacuated high or mixed density lesion >25cc not surgically evacuated)*<br>**6.** Other, please specify:	

v103, 61

Cardiac Arrest *(prior to ED or in hospital according to patient chart or TACPC ACR)*

1. Yes, 1 arrest
2. Yes, two arrests
3. Yes, >2 arrests, specify:
4. No (no documented cardiac arrest)
5. Other, please specify:

v104-v105 62-77

Cardiac Arrest 1 Date ____ ____ ____ ____ / ____ ____ / ____ ____ (y y y y m m d d)

Cardiac Arrest 1 Time ___ ___ : ___ ___ (24 hr) (h h m m)

v106-v107 78-93

Cardiac Arrest 2 Date ____ ____ ____ ____ / ____ ____ / ____ ____ (y y y y m m d d)

Cardiac Arrest 2 Time ___ ___ : ___ ___ (24 hr) (h h m m)

v108, 94

Respiratory Arrest *(prior to ED or in hospital according to patient chart or TACPC ACR)*

1. Yes, 1 arrest
2. Yes, two arrests
3. Yes, >2 arrests, specify:
4. No (no documented respiratory arrest)
5. Other, please specify:

109-v110 95-110

Respiratory Arrest 1 Date ____ ____ ____ ____ / ____ ____ / ____ ____ (y y y y m m d d)

Resp. Arrest 1 Time ___ ___ : ___ ___ (24 hr) (h h m m)

v111-v112 111-126

Respiratory Arrest 2 Date ____ ____ ____ ____ / ____ ____ / ____ ____ (y y y y m m d d)

Resp. Arrest 2 Time ___ ___ : ___ ___ (24 hr) (h h m m)

SURGICAL INTERVENTIONS

row 6 v113, 1-2

Number of Operative Visits *(hospital chart)*

_____ _____

v114, 3

Operation(s) *(hospital chart)*

1 = Yes / **2** = Not documented / **3** = No

↓ If *Yes, complete the TOPHR HIT* ***Operation Record Form (Form F)*** *for each operation and at the end of the patient stay please record the total number of operations below.*

v115, 4-5

Number of operations

---

[2] *a new classification of head injury based on computerized tomography – Journal of Neurosurgery Nov 1991 (75) S14 – S20*

VENTILATION

v116, 6

Ventilated *(according to ACR, 'Respiratory Therapy Record', and/or 'Nursing Notes'. Prehospital, ED, Hospital, and Trauma/Intensive Care Unit ventilation are all included. If ventilated more than three times, please complete a* ***TOPHR HIT Ventilation Form (Form G)*** *for each additional ventilation)*

1. Not ventilated
2. Ventilated Once
3. Ventilated Twice
4. Ventilated Three Times
5. Ventilated Four or More Times, specify:__ __ *(please use Form G for each additional ventilation)*
6. Not documented in ACR or Hospital Chart
7. Other, please specify:

v117-v118 7-38

Ventilated 1 Start Date/Time (00:00 (midnight) – 23:59 hrs)

__ __ __ __ / __ __ __ / __ __ __ __ : __ __
y y y y m m m d d h h m m

Ventilated 1 (or T-piece) Stop Date/Time

__ __ __ __ / __ __ __ / __ __ __ __ : __ __
y y y y m m m d d h h m m

v119-v120 39-70

Ventilated 2 Start Date/Time (00:00 (midnight) – 23:59 hrs)

__ __ __ __ / __ __ __ / __ __ __ __ : __ __
y y y y m m m d d h h m m

Ventilated 2 (or T-piece) Stop Date/Time

__ __ __ __ / __ __ __ / __ __ __ __ : __ __
y y y y m m m d d h h m m

v121, v122 71-102

Ventilated 3 Start Date/Time (00:00 (midnight) – 23:59 hrs)

__ __ __ __ / __ __ __ / __ __ __ __ : __ __
y y y y m m m d d h h m m

Ventilated 3 (or T-piece) Stop Date/Time

__ __ __ __ / __ __ __ / __ __ __ __ : __ __
y y y y m m m d d h h m m

OTHER INTERVENTIONS

row 7 v123, 1-5	**Packed Red Blood Cells Transfused** *(total first 24 hours from date & time of ED arrival. See Nurse's notes. SMH 350 mL/bag))*	____ ____ ____ ____ ____ mL
	**IV Fluid** *(total transfused from hospital chart during 1st 24 hours from date & time of ED arrival. See Nurse's notes)*	
v124, 6-10	**Albumin 5%**	____ ____ ____ ____ ____ mL
v125, 11-15	**Albumin 23%**	____ ____ ____ ____ ____ mL
v126, 16-20	**Fresh Frozen Plasma** *(SMH 250 mL/bag))*	____ ____ ____ ____ ____ mL
v127, 21-25	**Platelets** *(SMH 25 mL/unit)*	____ ____ ____ ____ ____ mL
v128, 26-30	**Pentaspan**	____ ____ ____ ____ ____ mL
v129, 31-35	**D5W**	____ ____ ____ ____ ____ mL
v130, 36-40	**Normal Saline**	____ ____ ____ ____ ____ mL
v131, 41-45	**Ringers Lactate**	____ ____ ____ ____ ____ mL
v132, 46-50	**D10**	____ ____ ____ ____ ____ mL
v133, 51-55	**2/3 1/3**	____ ____ ____ ____ ____ mL
v134, 56-60	**½ Normal Saline**	____ ____ ____ ____ ____ mL
v135, 61-65	**Study Fluid** *(hospital chart or TACPC ACR Clinical Information –CTPR and from TPDC)*	____ ____ ____ ____ ____ mL
v136, 66-70	**Mannitol**	____ ____ ____ ____ ____ mL

v137, 71-75 **Other IV Fluid, please specify:** ____ ____ ____ ____ ____ mL

v138, 76-80 **Total IV Fluid** *(received 1st 24 hours from date & time of ED arrival)* ____ ____ ____ ____ ____ mL

v139, 81 **Oxygen Therapy** *(any documented oxygen therapy from TACPC ACR Clinical Information – Treatment prior to arrival and ACR - CTPR (codes 130 – 142))*
1 = Yes / 2 = Not documented on ACR / 3 = No

v140, 82 **Immobilization** *(TACPC ACR Clinical Information – Treatment prior to arrival and ACR - CTPR (codes 105, 111, 112, 113, and 115))*
1 = Yes / 2 = Not documented on ACR / 3 = No

v141, 83 **Airway Management** *(airway management or airway breathing procedure from TACPC ACR Clinical Information – Treatment prior to arrival and ACR - CTPR (codes170, 171, 322, 324, and 326))*
1 = Yes / 2 = Not documented on ACR / 3 = No

v142, 84 **Ventilation** *(TACPC ACR Clinical Information – Treatment prior to arrival and ACR - CTPR (codes 143, 143))*

1. No ventilation since patient breathing spontaneously
2. Yes, manual assist bag valve mask (BMV)
3. Yes, automatic assist (e.g., Genesis)
4. Not documented on ACR
5. Other, please specify:

v143, 85 **Chest Decompression Needle / Thoracostomy** *(TACPC ACR Clinical Information – Treatment prior to arrival and ACR - CTPR (codes 320, 321))*

1. Yes (320)
2. Unsuccessful (321)
3. Both 320 and 321
4. Not documented on ACR
5. No
6. Other, please specify:

v144, 86 **Haemorrhage Control** *(TACPC ACR Clinical Information – Treatment prior to arrival and ACR - CTPR (code 101))*
1 = Yes / 2 = Not documented on ACR / 3 = No

LABORATORY

	Normal range = the lowest "low normal" and highest "high normal" from either SMH and SWCHSC	**Arrival ED** *(closest value to "Arrival to ED" (transcribe date/time from page 3))* _ _ _ _/_ _ _/ _ _ y y y y m m m d d __ __ : __ __ (24 hr) h h m m	**1.5 Hours** *(closest value to 1.5 hours post arrival ED (1-2 hours))* _ _ _ _/_ _ _/ _ _ y y y y m m m d d __ __ : __ __ h h m m	**12 Hours** *(post arrival ED and highest value ±1 hour (11 - 13 hours))* _ _ _ _/_ _ _/ _ _ y y y y m m m d d __ __ : __ __ h h m m	**24 Hours** *(post arrival ED and highest value ±1 hour (23 - 25 hours))* _ _ _ _/_ _ _/ _ _ y y y y m m m d d __ __ : __ __ h h m m	**48 Hours** _ _ _ _/_ _ _/ _ _ y y y y m m m d d __ __ : __ __ h h m m
row 8 v145, 1-4	**Random Glucose mmol/L** *(4.0-8.0 mmol/L)*	1.Available/ 2.Not Available ↓ ___ ___ . ___ mmol/L	1.Available/ 2.Not Available ↓ ___ ___ . ___ mmol/L	1.Available/ 2.Not Available ↓ ___ ___ . ___ mmol/L	1.Available/ 2.Not Available ↓ ___ ___ . ___ mmol/L	1.Available/ 2.Not Available ↓ ___ ___ . ___ mmol/L
v146, 5-6	**Alcohol Level** *(if 0 mmol/L at arrival to ED then do not input a value for 1.5 hours)*	1.Available/ 2.Not Available ↓ ___ ___ mmol/L	1.Available/ 2.Not Available ↓ ___ ___ mmol/L	1.Available/ 2.Not Available ↓ ___ ___ mmol/L	1.Available/ 2.Not Available ↓ ___ ___ mmol/L	1.Available/ 2.Not Available ↓ ___ ___ mmol/L
v147, 7-9	**HGB** *(115-180 g/L)*	1.Available/ 2.Not Available ↓ ___ ___ ___ g/L	1.Available/ 2.Not Available ↓ ___ ___ ___ g/L	1.Available/ 2.Not Available ↓ ___ ___ ___ g/L	1.Available/ 2.Not Available ↓ ___ ___ ___ g/L	1.Available/ 2.Not Available ↓ ___ ___ ___ g/L
v148, 10-13	**Blood Gases** **Arterial pH** *(7.35 – 7.45)*	1.Available/ 2.Not Available ↓ ____ . ____ ____pH	1.Available/ 2.Not Available ↓ ____ . ____ ____pH	1.Available/ 2.Not Available ↓ ____ . ____ ____pH	1.Available/ 2.Not Available ↓ ____ . ____ ____pH	1.Available/ 2.Not Available ↓ ____ . ____ ____pH
v149, 14-15	**Arterial PCO_2 mmHg** *(35-45 mmHg)*	____ ____ mmHg	____ ____ mmHg	____ ____ mmHg	____ ____ mmHg	____ ____ mmHg
v150, 16-	**Arterial PO_2**					

18	**mmHg** *(80-100 mmHg)*	___ ___ ___ mmHg	___ ___ ___ mmHg	___ ___ ___ mmHg	___ ___ ___ mmHg	___ ___ ___ mmHg
v151, 19-20	**Albumin** *(35 – 50 g/L)*	1.Available/ 2.Not Available ↓ ____ ____ g/L	1.Available/ 2.Not Available ↓ ____ ____ g/L	1.Available/ 2.Not Available ↓ ____ ____ g/L	1.Available/ 2.Not Available ↓ ____ ____ g/L	1.Available/ 2.Not Available ↓ ____ ____ g/L
v152, 21-23	**Serum Lactate** *(0.5 – 2.0 mmol/L)*	1.Available/ 2.Not Available ↓ ____ . ____ mmol/L	1.Available/ 2.Not Available ↓ ____ . ____ mmol/L	1.Available/ 2.Not Available ↓ ____ . ____ mmol/L	1.Available/ 2.Not Available ↓ ____ . ____ mmol/L	1.Available/ 2.Not Available ↓ ____ . ____ mmol/L
v153, 24-25	**Total Bilirubin µmol/L** *(<20 µmol/L)*	1.Available/ 2.Not Available ↓ _____ _____ µmol/L	1.Available/ 2.Not Available ↓ _____ _____ µmol/L	1.Available/ 2.Not Available ↓ _____ _____ µmol/L	1.Available/ 2.Not Available ↓ _____ _____ µmol/L	1.Available/ 2.Not Available ↓ _____ _____ µmol/L
v154, 26-28	**Serum Sodium mmol/L** *(135-147 mmol/L)*	1.Available/ 2.Not Available ↓ ___ ___ ___ mmol/L	1.Available/ 2.Not Available ↓ ___ ___ ___ mmol/L	1.Available/ 2.Not Available ↓ ___ ___ ___ mmol/L	1.Available/ 2.Not Available ↓ ___ ___ ___ mmol/L	1.Available/ 2.Not Available ↓ ___ ___ ___ mmol/L
v155, 29-31	**Serum Chloride mmol/L** *(96 - 108 mmol/L)*	1.Available/ 2.Not Available ↓ ___ ___ ___ mmol/L	1.Available/ 2.Not Available ↓ ___ ___ ___ mmol/L	1.Available/ 2.Not Available ↓ ___ ___ ___ mmol/L	1.Available/ 2.Not Available ↓ ___ ___ ___ mmol/L	1.Available/ 2.Not Available ↓ ___ ___ ___ mmol/L
v156, 32-34	**Total Calcium mmol/L** *(2.2-2.6 mmol/L)*	1.Available/ 2.Not Available ↓ ____ . ____ mmol/L	1.Available/ 2.Not Available ↓ ____ . ____ mmol/L	1.Available/ 2.Not Available ↓ ____ . ____ mmol/L	1.Available/ 2.Not Available ↓ ____ . ____ mmol/L	1.Available/ 2.Not Available ↓ ____ . ____ mmol/L
v157, 35-37	**Serum Potassium mmol/L** *(3.5 - 5.0 mmol/L)*	1.Available/ 2.Not Available ↓ ____ . ____ mmol/L	1.Available/ 2.Not Available ↓ ____ . ____ mmol/L	1.Available/ 2.Not Available ↓ ____ . ____ mmol/L	1.Available/ 2.Not Available ↓ ____ . ____ mmol/L	1.Available/ 2.Not Available ↓ ____ . ____ mmol/L
v158, 38-39	**Serum Bicarbonate mmol/L** *(13 - 30 mmol/L)*	1.Available/ 2.Not Available ↓ ____ ____ mmol/L	1.Available/ 2.Not Available ↓ ____ ____ mmol/L	1.Available/ 2.Not Available ↓ ____ ____ mmol/L	1.Available/ 2.Not Available ↓ ____ ____ mmol/L	1.Available/ 2.Not Available ↓ ____ ____ mmol/L
v159, 40-43	**Serum Magnesium mmol/L** *(0.63 – 1.10 mmol/L)*	1.Available/ 2.Not Available ↓ ___ . ___ ___ mmol/L	1.Available/ 2.Not Available ↓ ___ . ___ ___ mmol/L	1.Available/ 2.Not Available ↓ ___ . ___ ___ mmol/L	1.Available/ 2.Not Available ↓ ___ . ___ ___ mmol/L	1.Available/ 2.Not Available ↓ ___ . ___ ___ mmol/L
v160, 44-46	**Serum Osmolarity mmol/Kg** *(280-300 mmol/Kg)*	1.Available/ 2.Not Available ↓ ___ ___ ___ mmol/Kg	1.Available/ 2.Not Available ↓ ___ ___ ___ mmol/Kg	1.Available/ 2.Not Available ↓ ___ ___ ___ mmol/Kg	1.Available/ 2.Not Available ↓ ___ ___ ___ mmol/Kg	1.Available/ 2.Not Available ↓ ___ ___ ___ mmol/Kg
v161, 47	**Rouleau (S&W)** *(not documented = absent)*	1.Available/ 2.Not Available ↓ **1.** Absent **2.** Present **3.** Other, specify:	1.Available/ 2.Not Available ↓ **1.** Absent **2.** Present **3.** Other, specify:	1.Available/ 2.Not Available ↓ 1. Absent 2. Present 3. Other, specify:	1.Available/ 2.Not Available ↓ 1.Absent 2.Present 3.Other, specify:	1.Available/ 2.Not Available ↓ 1.Absent 2.Present 3.Other, specify:
v162, 48	**Rouleau (SMH)** *(not documented = absent)*	1.Available/ 2.Not Available ↓ **1.** Absent **2.** Present **3.** Other, specify:	1.Available/ 2.Not Available ↓ **1.** Absent **2.** Present **3.** Other, specify:	1.Available/ 2.Not Available ↓ **1.** Absent **2.** Present **3.** Other, specify:	1.Available/ 2.Not Available ↓ **1.** Absent **2.** Present **3.** Other, specify:	1.Available/ 2.Not Available ↓ **1.** Absent **2.** Present **3.** Other, specify:
v163, 49-52	**PTT** *(25.5 – 35.0)*	1.Available/ 2.Not Available ↓ ___ ___ . ___	1.Available/ 2.Not Available ↓ ___ ___ . ___	1.Available/ 2.Not Available ↓ ___ ___ . ___	1.Available/ 2.Not Available ↓ ___ ___ . ___	1.Available/ 2.Not Available ↓ ___ ___ . ___
v164, 53-56	**INR** *(0.81 - 1.2)*	1.Available/ 2.Not Available ↓ ___ . ___ ___	1.Available/ 2.Not Available ↓ ___ . ___ ___	1.Available/ 2.Not Available ↓ ___ . ___ ___	1.Available/ 2.Not Available ↓ ___ . ___ ___	1.Available/ 2.Not Available ↓ ___ . ___ ___

v165, 57-59	**Platelet Count (PLT)** *(140 – 400x10⁹/L)*	1.Available/ 2.Not Available ↓ ____ ____ ____ *10⁹/L*	1.Available/ 2.Not Available ↓ ____ ____ ____ *10⁹/L*	1.Available/ 2.Not Available ↓ ____ ____ ____ *10⁹/L*	1.Available/ 2.Not Available ↓ ____ ____ ____ *10⁹/L*	1.Available/ 2.Not Available ↓ ____ ____ ____ *10⁹/L*

HOSPITAL OUTCOME

row 9, v166, 1

Status at time of abstraction

1=Died in hospital *(see below)* / **2**=Survived to discharge ***(skip to next section)*** / **3**=Not doc. in chart / **4**=Still in / **5**=Other, specify:
↓

HOSPITAL OUTCOME – DIED INHOSPITAL

v167-v168, 2-17

Date of Death Inhospital

____ ____ ____ ____ / ____ ____ ____ / ____ ____
y y y y m m m d d

Time of Death Inhospital
((00:00 (midnight) – 23:59 hrs)

____ ____ : ____ ____ hours
h h m m

v169, 18

Unique Circumstances *(according to hospital chart doctor's notes)*

1. DNR – not resuscitated (passive)
2. Withdrawal of treatment (active)
3. Not documented in chart
4. Withheld CPR
5. Withdrawal CPR
6. Other, please specify:

v170, 19-49

Immediate Cause of Death a) *(according to 'Cause of Death' section from the 'Medical Certificate of Death')* ____________________

v171, 50-69

Due To, or as a consequence of, b) ____________________

v172, 70-84

c) ____________________

v173, 85-104

d) ____________________

v174, 105-124

Other significant conditions contributing to the death but not causally related to the *immediate cause (a)* above ____________________

v175, 125

Autopsy

1 = Yes / **2** = Not documented / **3** = No
↓ If *Yes,* please specify autopsy details:

<Skip to "HOSPITAL OUTCOME - Discharge or Death (Inhospital) or Patient Withdrawal">

HOSPITAL OUTCOME – SURVIVED TO DISCHARGE

row 10 v176,-v177, 1-16

Date of Discharge *(Note: all of those who survived to discharge will be asked back for testing at 4 and 12 months by the Inhospital Research Assistant. The Inhospital Research Assistant is responsible for booking and overseeing the 6 month and 12 month tests in collaboration with the Research Assistant (Paula). The Inhospital Research Assistant is also responsible to a reminder phone call at 2 months post discharge and again at 1 week prior to the month 4 and month 12 appointments. Call logs must be forwarded to the Study Administrator Vincenza soon after contact with a patient or patient representative is made)*

____ ____ ____ ____ / ____ ____ ____ / ____ ____
y y y y m m m d d

Time of Discharge (00:00 (midnight) – 23:59 hrs)

____ ____ : ____ ____ hours
h h m m

v178, 17

Discharged to *(from hospital chart 'Discharge Notes')*

1. Home
2. Non acute extended care facility (e.g., rehabilitation facility), *please fill in Form A*
3. Non acute chronic care facility (e.g., nursing home), *please fill in Form A*

4. Left against medical advice (AMA)
5. Another acute care hospital, *please fill in Form A*
6. Not documented in the chart
7. Other, please specify:

v179, 18

Cerebral Performance Score (CPS)[3] *(Inhospital Research Assistant to administer at discharge. If not possible, inhospital Research Assistant to derive from 'Discharge Notes')*

1. **Good cerebral performance** *(i.e., conscious, alert, normal cerebral function. May have minor psychologic or neurologic deficits that do not significantly compromise cerebral of physical function*
2. **Moderate cerebral disability** *(i.e., conscious alert, sufficient cerebral function for activities of daily life (e.g., dress, travel by public transportation, food preparation). May have hemiplegia, seizures, ataxia, dysarthria, dysphasia, or permanent memory or mental changes)*
3. **Severe cerebral disability** *(i.e., conscious has at least limited cognition. Dependent on others for daily support (i.e., in an institution or at home with exceptional family effort) because of impaired brain function. Includes wide range of cerebral abnormalities, from ambulatory patients who have severe memory disturbance or dementia precluding independent existence, to paralyzed patients who can communicate only with their eyes, as in the locked-in syndrome)*
4. **Coma, vegetative state** *(i.e., not conscious, unaware of surroundings, no cognition. No verbal and/or psychologic interaction with environment. May appear awake because of spontaneous eye opening or sleep awake cycle. Includes all degrees of unresponsiveness that are neither CPC 3 (conscious) nor CPC 5 (coma that satisfies brain death criteria).*
5. **Brain Death** *(with beating heart) or death (without beating heart) Apnea, areflexia, "coma" electroencephalographic silence.*
   CP A. Anesthesia (CNS depressants)
   Uncertain as to above categories because of anesthetic, other CNS depressant drug or relaxant effects.

HOSPITAL OUTCOME - SURVIVED TO DISCHARGE - ABBREVIATED INJURY SCALE (AIS)[4]

v180,- v181 19-34

Abbreviated Injury Scale (AIS) Date *(Inhospital Research Assistant to administer at discharge. If not possible, transcribe from 'Physiotherapy Notes')*

____ ____ ____ ____ / ____ ____ ____ / ____ ____
y y y y m m m d d

AIS Time (00:00 (midnight) – 23:59 hrs)

____ ____ : ____ ____ hours
h h m m

v182, 35	**Head**	**1. None / 1. Minor / 2. Moderate / 3. Severe not life-threatening / 4. Severe life-threatening / 5. Critical / 6. Unsurvivable**
v183, 36	**Face**	**1. None / 1. Minor / 2. Moderate / 3. Severe not life-threatening / 4. Severe life-threatening / 5. Critical / 6. Unsurvivable**
v184, 37	**Neck**	**1. None / 1. Minor / 2. Moderate / 3. Severe not life-threatening / 4. Severe life-threatening / 5. Critical / 6. Unsurvivable**
v185, 38	**Chest**	**1. None / 1. Minor / 2. Moderate / 3. Severe not life-threatening / 4. Severe life-threatening / 5. Critical / 6. Unsurvivable**
v186, 39	**Abdomen**	**1. None / 1. Minor / 2. Moderate / 3. Severe not life-threatening / 4. Severe life-threatening / 5. Critical / 6. Unsurvivable**
v187, 40	**Spine**	**1. None / 1. Minor / 2. Moderate / 3. Severe not life-threatening / 4. Severe life-threatening / 5. Critical / 6. Unsurvivable**
v188, 41	**Upper Limb**	**1. None / 1. Minor / 2. Moderate / 3. Severe not life-threatening / 4. Severe life-threatening / 5. Critical / 6. Unsurvivable**
v189, 42	**Lower Limb (inc. pelvis)**	**1. None / 1. Minor / 2. Moderate / 3. Severe not life-threatening / 4. Severe life-threatening / 5. Critical / 6. Unsurvivable**
v190, 43	**External**	**1. None / 1. Minor / 2. Moderate / 3. Severe not life-threatening / 4. Severe life-threatening / 5. Critical / 6. Unsurvivable**

HOSPITAL OUTCOME - SURVIVED TO DISCHARGE – DISABILITY RATING SCALE (DRS)[5]

row 11 v191,- v192, 1-16

Disability Rating Scale (DRS) Date *(the Inhospital Research Assistant to administer at discharge. If not possible, transcribe from 'Physiotherapy Notes'. Please complete the* ***TOPHR HIT Disability Rating Scale (DRS) Form (Form H)*** *and transcribe below)*

____ ____ ____ ____ / ____ ____ ____ / ____ ____
y y y y m m m d d

DRS Time (00:00 (midnight) – 23:59 hrs)

____ ____ : ____ ____ hours
h h m m

---

[3] Safar P., Bircher N. Cardiopulmonary Cerebral Resuscitation, Guidelines by the World Federation of Societies of Anesthesiologists (WFSA). 3rd edition. Philadelphia WB Saunders 1998.
[4] Dick W. and Baskett (1999) Acta Anaesthesiologica Belgica, 2000, 51, no. 1, 18-38
[5] Rappaport M., et al. (1982) Arch Phys Med Rehabil, 63, March, 118 – 123 & http://www.tbims.org/combi/drs/index.html (date last accessed Dec 3, 2004)

v193 17-18

Disability Rating Scale (DRS) Score *(please obtain the total score by completing the **TOPHR HIT Disability Rating Scale Form (Form H)**)*

_____ _____

HOSPITAL OUTCOME - SURVIVED TO DISCHARGE – FUNCTIONAL INDEPENDENCE MEASURE (FIM)[6]

v194- v195 19-34

Functional Independence Measure (FIM) Date

___ ___ ___ ___ / ___ ___ ___ / ___ ___
y y y y m m m d d

FIM Time (00:00 (midnight) – 23:59 hrs)

___ ___ : ___ ___ hours
h h m m

v196 35-36

Functional Independence Measure (FIM) Score *(please obtain the total score by completing the **TOPHR HIT Functional Independence Measure (FIM) Form (Form J)**)*

_____ _____

HOSPITAL OUTCOME - DISCHARGE OR DEATH INHOSPITAL OR PATIENT WITHDRAWAL

v197, 37

Adverse Drug Reaction (ADR)
*(any mention anywhere of any known or suspected ADR to the study fluid defined as "all noxious and unintended responses to a medicinal product related to any dose"[7]. A **separate TOPHR HIT Adverse Drug Reaction Form (Form I)** must be completed for each known or suspected ADR(s) to the study fluid)*

1 = Yes *(Please complete **Form I**)* / **2** = No / **3** = Other, please specify:

198, 38

Patient Alive at 24 hours *(the difference between date/time inhospital patient death or discharge and date/time of arrival to ED ≥24 hours)*

1 = Yes / **2** = No / **3** = Other, please specify:

v199, 39

Patient Alive at 48 hours *the difference between date/time inhospital patient death or discharge and date/time of arrival to ED ≥48 hours)*

1 = Yes / **2** = No / **3** = Other, please specify:

v200, 40

Glasgow Outcome Scale (GOS)[8] *(please complete **TOPHR HIT Case Report Form – Inhospital GOS Form (Form K)**)*

1. **Death**
2. **Vegetative State (VS)**
3. **Severe Disability (SD)**
4. **Moderate Disability (MD)**
5. **Good Recovery (GR)**

v201, 41

Glasgow Outcome Scale – Extended (GOS – E)[9] *(please complete **TOPHR HIT Case Report Form – Inhospital GOS - E Form (Form L)**)*

1. **Death**
2. **Vegetative State (VS)**
3. **Lower Severe Disability (Lower SD)**
4. **Upper Severe Disability (Upper SD)**
5. **Lower Moderate Disability (Lower MD)**
6. **Upper Moderate Disability (Upper MD)**

---

[6] Dodds, A., et al. (1993) Arch Phys Med Rehabil, 74, May, 531 – 536 & http://www.tbims.org/combi/drs/index.html (date last accessed Dec 3, 2004)
[7] Guidance for Industry: Clinical Safety Data Management Definitions and Standards for Expedited Reporting ICH Topic E2A, Health Products and Food Branch Guidance Document, Health Canada - Publications, 1995 Catalogue No. H42-2/67-8-1995E page 2
[8] Journal of Neurotrauma (1998) Lindsay J. T. et al. 15, 8, 573 - 585
[9] Journal of Neurotrauma (1998) Lindsay J. T. et al. 15, 8, 573 - 585

7. **Lower Good Recovery (Lower GR)**

8. **Upper Good Recovery (Upper GR)**

row 12 v202, 1-80

Most responsible diagnosis at discharge or death *as written in the text column by the Health Records/Data Department in the 'Discharge Summary Sheet' or 'Discharge and Notification Form')*

___ ___ ___ ___ ___ ___ ___ ___ ___ ___ ___ ___ ___ ___ ___ ___ ___ ___ ___ ___

row 13 v203, 1-80

Primary diagnosis / comorbid condition at discharge or death *(as written in the text column by the Health Records/Data Department in the 'Discharge Summary Sheet' or 'Discharge and Notification Form')*

1) ___

row 14 v204, 1-80

2) ___

row 15 v205, 1-80

3) ___

RESEARCH COORDINATOR ABSTRACTION DETAILS

This form is highly confidential. Access to this form must be limited to the Saint Michael's Hospital (SMH) or the Sunnybrook & Women's College Health Sciences Centre (SWCHSC) Trauma Inhospital Research Assistants. When not in use, this form must be locked in a secure location. Upon completion, please ensure that all fields have been addressed and are accurate and notify Tyrone Perreira, Paramedic Research Coordinator, Prehospital and Transport Medicine Research Program, Room B103, Sunnybrook & Women's College Health Science Centre (SWCHSC) Tel: 416-480 6100x7072 Email: tyrone.perreira@sw.ca. Tyrone will make arrangements for a hand-to-hand pick-up of the forms. The appropriate Trauma Inhospital Research Assistant, Paramedic Coordinator, and Principle Investigator (PI) must initial or sign and date this form prior to data entry. Any changes to this form post PI signature must be documented with the ***TOPHR HIT Field Revision Form****. All revisions must be signed and dated by the person responsible for the change and by the PI.*

row 16 v206, 1-2

Inhospital Abstractor Initials ______ ______ (first / last)

v207, 3-4

Paramedic Research Coordinator Initials: ______ ______ (first / last)

v208, 5-15

Date Paramedic Research Coordinator Review: ____ ____ ____ ____ / ____ ____ ____ / ____ ____ (y y y y / m m m / d d)

v209, 16

Subject ID Code Assignment *(Subject ID Code is assigned by the TOPHR HIT Paramedic Research Coordinator (TP) for cases that meet the study criteria)*

1 = Yes / **2** = No / **3** = Other, specify:

↓ If *Yes,*

v210, 17-19

Subject ID Code *(assigned by the TOPHR HIT Paramedic Research Coordinator (TP) Paramedic Research Coordinator for cases that meet study criteria)*

______ ______ ______

INVESTIGATOR STATEMENT

I certify that: I have carefully examined and verified all entries in this case form. All information entered onto these forms by myself and/or my associates is correct.

Principle Investigator Signature (LJM): ____________________________________

Date of Principle Investigator Verification (LJM): ____ ____ ____ ____ / ____ ____ ____ / ____ ____ (y y y y / m m m / d d)

TOPHR HIT CASE REPORT FORM – INHOSPITAL, FINAL VERSION 24 MAR 2005 SUPPLEMENTARY FORMS:

1. TOPHR HIT TRAUMA/INTENSIVE CARE SETTING FORM (FORM B)
2. TOPHR HIT APACHE II FORM (FORM C)
3. TOPHR HIT SOFA FORM (FORM D)
4. TOPHR HIT MODS FORM (FORM E)
5. TOPHR HIT OPERATION RECORD FORM (FORM F)
6. TOPHR HIT VENTILATION FORM (FORM G)
7. TOPHR HIT DISABILITY RATING SCALE (DRS) (FORM H)
8. TOPHR HIT ADVERSE DRUG REACTION FORM (FORM I)
9. TOPHR HIT FUNCTIONAL INDEPENDENCE MEASURE (FIM) FORM (FORM J))
10. TOPHR HIT GOS FORM (FORM K)
11. TOPHR HIT CASE REPORT FORM – INHOSPITAL GOS - EXTENDED FORM (FORM L)
